# Quadruple ultrasound, photoacoustic, optical coherence, and fluorescence fusion imaging with a transparent ultrasound transducer

**DOI:** 10.1073/pnas.1920879118

**Published:** 2021-03-08

**Authors:** Jeongwoo Park, Byullee Park, Tae Yeong Kim, Sungjin Jung, Woo June Choi, Joongho Ahn, Dong Hee Yoon, Jeongho Kim, Seungwan Jeon, Donghyun Lee, Uijung Yong, Jinah Jang, Won Jong Kim, Hong Kyun Kim, Unyong Jeong, Hyung Ham Kim, Chulhong Kim

**Affiliations:** ^a^School of Interdisciplinary Bioscience and Bioengineering, Pohang University of Science and Technology, 37673 Pohang, Republic of Korea;; ^b^Medical Device Innovation Center, Pohang University of Science and Technology, 37673 Pohang, Republic of Korea;; ^c^Department of Creative IT Engineering, Pohang University of Science and Technology, 37673 Pohang, Republic of Korea;; ^d^Department of Materials Science and Engineering, Pohang University of Science and Technology, 37673 Pohang, Republic of Korea;; ^e^School of Electrical and Electronics Engineering, Chung-Ang University, 06974 Seoul, Republic of Korea;; ^f^Department of Ophthalmology, School of Medicine, Kyungpook National University, 41944 Daegu, Republic of Korea; ^g^Department of Mechanical Engineering, Pohang University of Science and Technology, 37673 Pohang, Republic of Korea;; ^h^Department of Chemistry, Pohang University of Science and Technology, 37673 Pohang, Republic of Korea;; ^i^Department of Electrical Engineering, Pohang University of Science and Technology, 37673 Pohang, Republic of Korea

**Keywords:** transparent ultrasound transducer, optical imaging, ultrasound imaging, multimodal imaging

## Abstract

Ultrasound and optical imagers are used widely in a variety of biological and medical applications. In particular, multimodal implementations combining light and sound have been actively investigated to improve imaging quality. However, the integration of optical sensors with opaque ultrasound transducers suffers from low signal-to-noise ratios, high complexity, and bulky form factors, significantly limiting its applications. Here, we demonstrate a quadruple fusion imaging system using a spherically focused transparent ultrasound transducer that enables seamless integration of ultrasound imaging with photoacoustic imaging, optical coherence tomography, and fluorescence imaging. As a first application, we comprehensively monitored multiparametric responses to chemical and suture injuries in rats’ eyes in vivo, such as corneal neovascularization, structural changes, cataracts, and inflammation. As a second application, we successfully performed multimodal imaging of tumors in vivo, visualizing melanomas without using labels and visualizing 4T1 mammary carcinomas using PEGylated gold nanorods. We strongly believe that the seamlessly integrated multimodal system can be used not only in ophthalmology and oncology but also in other healthcare applications with broad impact and interest.

## Significance

Multimodal imaging based on optics and ultrasound can provide guide images and complementary structural and functional information, thus improving the accuracy of medical diagnosis and treatment monitoring. However, because conventional ultrasound transducers are opaque, in multimodal imaging with optics, the optical devices must be placed off-axis from the ultrasound transducer. This off-axis arrangement is prone to misalignment, adds complexity and bulk to the system, and can result in a low signal-to-noise-ratio. Here, we present a transparent ultrasound transducer at the heart of a quadruple fusion imaging system that seamlessly integrates ultrasound imaging, photoacoustic imaging, optical coherence tomography, and fluorescence imaging, and we demonstrate the system’s use in imaging responses to both ophthalmologic injuries and oncologic diseases.

Imaging methods using ultrasound (US) or light have been used widely in a variety of applications (1–3). In particular, US imaging (USI) is used extensively in various medical imaging fields, providing noninvasive to minimally invasive diagnosis (4–6). Even though USI provides functional information, such as blood flow or stiffness, this information is still limited (7). On the other hand, optical imaging (OI) systems can provide detailed structural and functional information on scales ranging from molecules to organs (8–10). Still, most OI is severely handicapped by its shallow penetration depth in turbid media (11).

Many studies have combined conventional USI and OI systems to capitalize on their complementary advantages while minimizing their drawbacks (12–26). For example, a combined photoacoustic imaging (PAI) and USI system can simultaneously provide functional information (e.g., hemoglobin oxygen saturation and blood flow) and structural information (e.g., the location and size of blood vessels) (12, 13). In addition, in a system combining optical coherence tomography (OCT) and USI, OCT mainly images microscopic structures and functions of tissues at depths within a couple of millimeters, whereas USI, because it is based on acoustic phenomena, provides similar image information in deep tissues (14). As another example, the integration of fluorescence imaging (FLI) and USI simultaneously provides accurate anatomical US images and a disease-specific and fluorescence (FL)-sensitive image (15). Beyond such dual modalities systems, a variety of triple-modal imaging systems, including USI/OCT/FLI, USI/OCT/PAI, and PAI/OCT/FLI, have been actively explored to provide multiparametric features of biological tissues in vivo (23–26).

While integrating USI and OI systems offers many advantages, seamless integration in a relatively simple and reliable way has been a challenge (27, 28). Because a traditional US transducer (UT) is opaque, the optical sensor and UT cannot be placed along the same axis (29). The resulting off-axis placement is disadvantageous to imaging in two ways: 1) To acquire optical and US images from the same region of interest (ROI), a bypass is needed for either the light or the US signal. The bypass increases the size, complexity, and cost of the sensor. 2) Since the optical sensor and UT are off-axis in a single housing, the coregistered images could be misaligned. The misalignment creates heterogeneity and a low signal-to-noise-ratio (SNR) in optical and US images. Recently, several research teams have explored using a transparent UT (TUT) to seamlessly integrate USI and OI. Poly(vinylidene fluoride)-based and lithium niobate (LNO)-based TUTs were the most commonly used piezoelectric materials (30–35). In particular, to obtain an in vivo photoacoustic (PA) image, Chen et al. (34) developed an unfocused LNO-based TUT with a high level of transparency (∼90%) and a high center frequency (37 MHz). However, due to the limitation of the unfocused UT, the quality of the PA image was not comparable to conventionally obtained results. Park et al. (35) also applied an LNO-based TUT to photoacoustically image a mouse tail in vivo. However, this TUT was limited in obtaining high-resolution PA images by its relatively low transparency (∼66%) and center frequency (11 MHz). In both studies, the SNRs were not sufficient for high-contrast PAI, and only the feasibility of PAI was investigated. Other groups developed transparent capacitive micromachined ultrasonic transducers (cMUTs) with 80 to 82% transparency (36, 37). However, owing to the well-known fundamental issues of cMUT, such as low US intensity or high biasing voltage requirements (38), it is still difficult to apply cMUT to practical applications.

In this study, we present a practical LNO-based single-crystal TUT and a quadruple fusion imaging system for ophthalmologic and oncologic applications that seamlessly integrates USI, PAI, OCT, and FLI via the TUT. The TUT is manufactured with transparent layers, including the electrodes, piezoelectric layer, matching layer (ML), and backing layer (BL). A spherical acoustic focusing lens allows us to achieve a high lateral resolution and SNR. Further, the quadruple fusion imaging system advantageously combines two or three imaging techniques, depending on disease models, without changing the system geometry. Using the quadruple fusion imaging system, we successfully monitored ophthalmic diseases in rat models in vivo. Specifically, after chemical and suture injuries, the eye simultaneously exhibits vascular disorders (corneal neovascularization [CNV]), structural changes, fibrous membrane induced by anterior chamber reaction, edema caused by inflammation, and cataracts (39–45). CNV, in particular, forms the basis of several visual pathologies that constitute the fourth most common cause of blindness worldwide (46). Thus, comprehensive observation using a multimodal imaging system offers clear benefits. Several imaging techniques, such as slit-lamp biomicroscopy, FLI, and OCT, have been used to monitor the anterior segment, including CNV. However, no multimodal imaging system has been developed to comprehensively monitor all the above-mentioned disorders. Our quadruple fusion imaging system is expected to open diagnostic and monitoring avenues: PAI and OCT angiography (OCTA) for CNV; USI for cataracts; OCT for structural changes, including edema and fibrous membrane; and FLI for inflammation.

As a second application, we imaged tumor-bearing mice (e.g., one group with melanomas and another group with 4T1 mammary carcinomas), using the multimodal imaging system without and with contrast agents, respectively. Melanoma is the fifth most common cancer in the United States and has the highest mortality rate among skin cancers (47–49). In particular, because the staging and treatment methods of the melanoma are determined according to its thickness, there has been a long-standing clinical need to accurately measure the melanoma thickness noninvasively (50). As another cancer model, 4T1 mammary breast cancers are widely used because of their advantages in making experimental animal models of human breast cancer (51). Hence, a comprehensive imaging analysis of a 4T1 tumor can speed successful bench-to-bedside translation for human breast cancer treatment (52). PAI is an imaging modality that excels in label-free functional imaging (e.g., hemoglobin oxygen saturation [sO_2_]) and contrast-enhanced molecular imaging. By observing pigmented cancers, the PAI function in our multimodal imaging system can provide label-free boundary detection of melanomas and their sO_2_ map. By analyzing agent-labeled nonpigmented cancers, it can map 4T1 carcinomas. For both types of cancers, USI provides the cancer boundaries in all dimensions and OCT provides detailed skin structures.

## Results

### TUT.

**Fig. 1.**
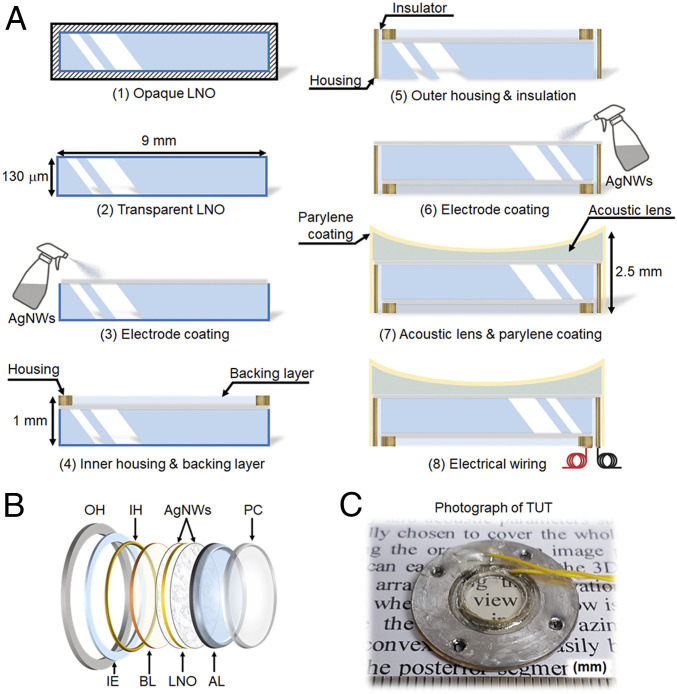
Fabrication process and assembly of a TUT. (*A*) The step-by-step fabrication process of a TUT. (*B*) Schematic illustration of the layer-by-layer TUT and enlarged view explaining each component. The center part of all components is transparent. (*C*) Photograph demonstrating the transparency of a TUT with an element size of 9 mm.

The eight-step fabrication process of the TUT, illustrated in Fig. 1*A*, is as follows.

1) A 36° rotated Y-cut LNO single crystal (Boston Piezo-Optics, Inc.) was selected as the piezoelectric material due to its high thickness-mode electromechanical coupling coefficient ($k_{t}$ ≈ 0.49), low dielectric permittivity ($\varepsilon_{s}$ ≈ 28), high longitudinal sound speed ($c_{l}$ ≈ 7,340 m/s), and high Curie temperature ($T_{C}$ ≈ 1,150 °C) (53).
2) A 9-mm-diameter disk-shaped LNO was transparently lapped to a thickness of 130 µm using a polisher system (Metprep 3TM Grinding/Polishing system, Allied High Tech).
3) As transparent electrodes, silver nanowires (AgNWs) were spray-coated on the surface of the polished LNO plate.
4) The inner housing (IH) was machined from brass and attached to the AgNWs side using a conductive epoxy (E-solder 3022, Von Roll) as an electrical bridge. After the IH was attached, degassed nonconductive epoxy (Epo-tek 301, Epoxy Technology Inc.) that acted as a BL was filled inside the IH to the same height as the IH. The BL was then cured for 24 h at room temperature. The epoxy used as the BL has the advantages of high transparency and a low acoustic impedance. In addition, the IH and BL were thin, with a 1-mm height that maximized light transmittance.
5) The outer housing (OH) was made of stainless steel. The OH was electrically isolated from the IH by using nonconductive insulation epoxy (IE).
6) The opposite LNO surface was spray-coated with AgNWs.
7) The acoustic impedance mismatch between the AgNWs-coated LNO (34.0 MRayls) and biological tissue (1.6 MRayls) was reasonably overcome by using a planoconcave acoustic lens (AL) and a parylene coating layer (PL) as the two MLs. As the first ML, the AL was properly lapped to minimize acoustic signal loss (54), and it also served to focus the US beam. For the PL second ML, a 21-µm-thick layer of parylene of was vapor-deposited on the entire TUT assembly using a parylene coater (PDS 2010, Specialty Coating Systems. Inc.). The PL not only minimized acoustic signal loss but also protected the exterior of the TUT.
8) Finally, the IH and the OH were wired separately using stranded cables.

The fully functional single-element TUT is shown in Fig. 1 *B* and *C*. Its layers are all optically transparent. The transparency of the TUT is demonstrated by photographing printed letters though the TUT. All materials and structures of the TUT were designed to achieve optimal performance through a Krimholtz−Leedom−Mattaei model simulation tool (PiezoCAD, Sonic Concepts, Inc.; *SI Appendix*, *Method S1*).

### Acoustic and Optical Properties of TUT.

**Fig. 2.**
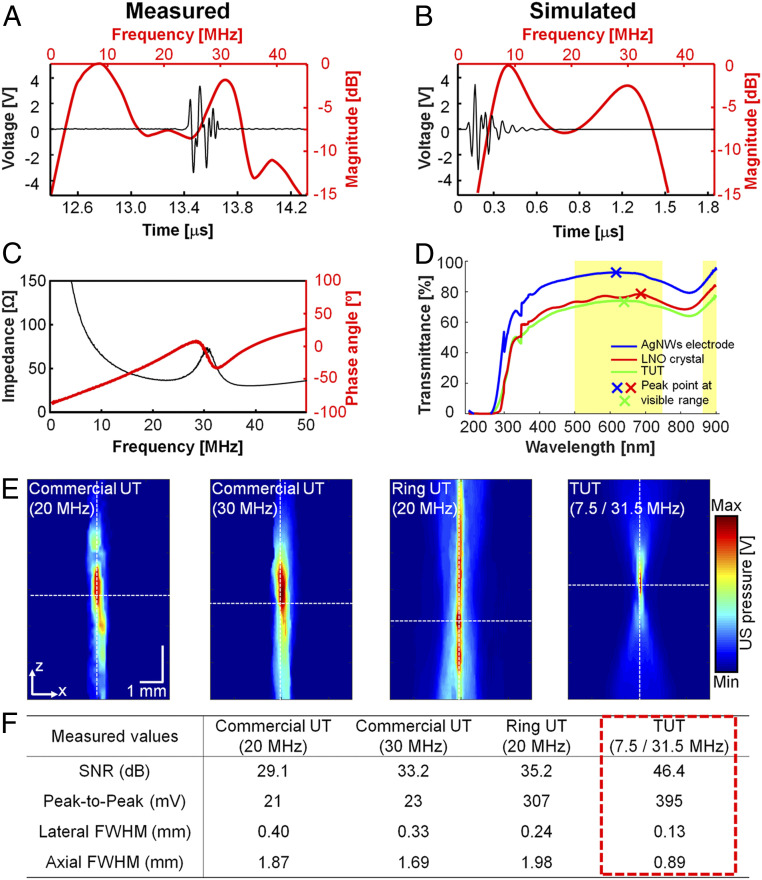
Acoustic and optical properties of a TUT. (*A*) Measured pulse echo response and frequency spectra, with a spatial pulse length of ∼0.1 μs and dual center frequencies (∼7.5 and ∼31.5 MHz) and bandwidths (∼13 MHz and 8 MHz). (*B*) Simulated pulse echo waveform and spectrum of the TUT in both the time and frequency domains. (*C*) Measured electrical impedance (53 Ω) and phase angle (−50°) spectra of the TUT, showing a good thickness-mode electromechanical coupling coefficient (0.68). (*D*) Light transmittance in the optical range from 200 nm to 900 nm. The yellow window represents a range of over 70% transparency of the TUT. (*E*) Comparison of the acoustic pressure fields of commercial UTs (20 and 30 MHz), a custom-made ring UT (20 MHz), and the TUT, measured by a hydrophone. All UTs are spherically focused. (*F*) Summary of SNR, peak-to-peak voltages, and lateral and axial FWHM.

To evaluate the performance of the TUT, we performed a series of measurements. In a pulse echo test, a dual-frequency feature in the spectral domain and a slightly long ring down in the time domain waveform were observed, which we attributed to the acoustically light damping material of the BL (Fig. 2*A*). The TUT had dual −6 dB center frequencies: a low frequency at 7.5 MHz and a high frequency at 31.5 MHz. The measured results matched well with simulation results (Fig. 2*B*). The black line shows that the TUT has an electrical impedance of 53 Ω and a phase angle of 8° at 31.5 MHz. These values match well with the 50 Ω input electrical impedance between the TUT and a pulser−receiver (P/R), enabling maximum power transfer (Fig. 2*C*). The optical transmittances of the AgNWs electrode, the LNO crystal, and the assembled TUT were measured at wavelengths from 200 nm to 900 nm (Fig. 2*D*). The AgNWs electrode exhibits its highest transparency of 92.5% at 620 nm. The LNO crystal achieves 78% transparency at around 680 nm, which matches well with the LNO’s intrinsic optical property (31). The assembled TUT has a peak transparency of 74% at 630 nm. The yellow bands in Fig. 2*D* indicate wavelengths where the transparency of the TUT is above 70%. Next, we compared the acoustic pressure fields of two commercial UTs with center frequencies of 20 MHz and 30 MHz, a custom-made ring UT (20 MHz), and the TUT (Fig. 2 *E* and *F*). All four UTs were spherically focused. The acoustic pressure field measured with the TUT is more tightly focused than those measured with the other UTs. The quantified SNR of the TUT is more than 11 dB higher than the highest value of the other transducers. The peak-to-peak voltage of the TUT is ∼15 to 17 times higher than the best value for the commercial UTs, and it is 1.3 times higher than that of the ring UT. Note that, despite the high peak-to-peak voltage of the ring UT, its SNR is relatively low due to the strong side-lobe signals of the ring-shaped beam pattern. The spectra of the commercial UTs and ring UT are shown in *SI Appendix*, Fig. S1*A*. The lateral and axial beam profiles of the respective UTs were acquired along the white dashed lines in Fig. 2*E* and are shown in *SI Appendix*, Fig. S1*B*. The TUT forms the tightest acoustic focus: Its focal point spreads are 1.8 to 3.1 times smaller than the others in the lateral direction and about 2 times smaller in the axial direction. These results strongly suggest that, among comparable alternatives, the TUT can achieve the highest SNR by focusing the most acoustic energy on the tiniest focal point.

### Seamlessly Integrated Quadruple Fusion Imaging System.

To show the geometrical advantage using the TUT, we compared various geometries of coaxial optical and acoustical alignments for multimodal imaging (*SI Appendix*, Fig. S2). A special opto-ultrasound beam combiner is widely used when a commercial UT is used for high-SNR reflection-mode PAI (*SI Appendix*, Fig. S2*A*). This approach inherently suffers from unavoidable acoustic attenuation and reflection in the beam combiner, resulting in signal losses. Moreover, the long acoustic and optical paths result in relatively low numerical apertures (NAs). To mitigate the above effects, a ring UT with a center hole allowing light transmission has been introduced (*SI Appendix*, Fig. S2*B*). However, there is a trade-off among the size of the hole, the acoustic sensitivity, and the optical NA. Further, the axial resolution of the ring UT can be deteriorated due to the Bessel-like acoustic field. All the problems mentioned above can be easily solved when the optical beam is transmitted through the TUT, a very simple and compact structure (*SI Appendix*, Fig. S2*C*).

**Fig. 3.**
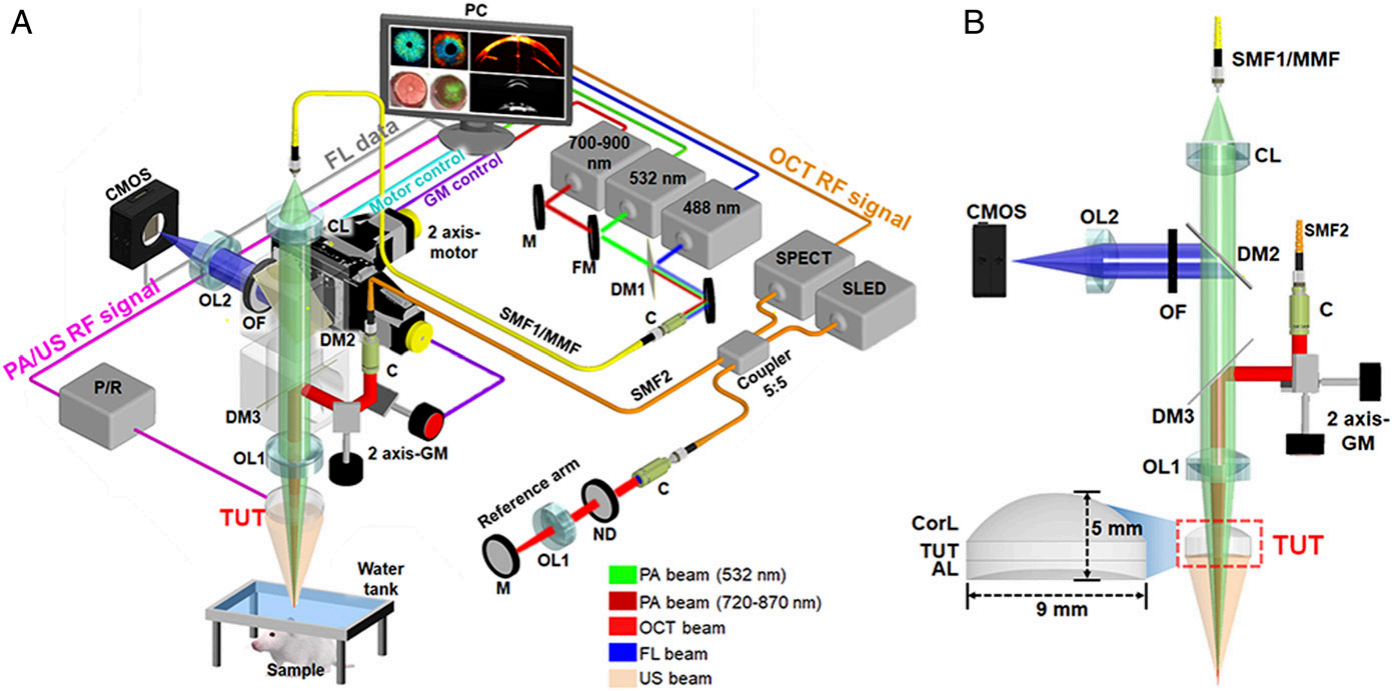
Schematic diagrams of a seamlessly integrated quadruple fusion imaging system using a TUT: USI, OCT, and FLI. (*A*) Overall schematic. (*B*) Magnified schematic of the imaging head module. PC, personal computer; RF, radio frequency; M, mirror; FM, flipping mirror; ND, neutral density filter; C, collimator; CorL, correction lens.

Fig. 3 is a schematic of the quadruple fusion imaging system that uses the TUT to seamlessly combine: USI, PAI, OCT, and FLI. There are eight key features of this fusion imaging system: 1) The 532-nm pulsed laser source for the PAI and the 488-nm continuous-wave (CW) laser source for the FLI are coupled to the same single-mode fiber (SMF1) through the first dichroic mirror (DM1). This 532-nm PAI wavelength is mainly used for ophthalmic applications. For oncologic applications, we use both the 532-nm laser for imaging vasculatures and a wavelength-tunable laser whose wavelength can be tuned between 700 nm and 900 nm for imaging tumors. The wavelength-tunable laser is coupled to a multimode fiber (MMF). SMF1 and MMF can be manually switched for each purpose. 2) The laser outputs from the SMF1 or the MMF are collimated and focused through a collimation lens (CL) and an objective lens (OL1) coaxially located with the TUT, and finally passed through the TUT to irradiate the sample. 3) The second DM (DM2) is in the path when performing FLI, but it is removed when performing PAI. 4) Three-dimensional (3D) PA and US signals are detected by the TUT with *x*–*y* raster scanning using two-axis mechanical motors. 5) When performing PAI or USI, a water tank with a bottom opening, covered with an optically and acoustically transparent membrane, is placed between the sample and the TUT. 6) The emitted FL signals are reflected by the DM2 and delivered to a complementary metal oxide semiconductor (CMOS) camera. 7) Light from a superluminescent light-emitting diode (SLED) OCT light source with a central wavelength of 860 nm is reflected 90° by the third DM (DM3) to achieve coaxial alignment with the TUT. 8) Optical scanning using two two-axis galvanometers (GMs) is performed to acquire 3D OCT data. More details of the system’s implementation and operation are provided in *Materials and Methods*.

### Comparative Performance of the Quadruple Fusion and Conventional Individual Imaging Systems.

We compared the system performance of our quadruple fusion imaging system with that of conventional individual imaging systems. First, the beam quality of the focused 532-nm light source was measured using a beam profiler (*SI Appendix*, Fig. S3*A*). The results confirmed that the beam qualities with and without the TUT were almost identical (*SI Appendix*, Fig. S3*B*). With no TUT in the beam path, the full widths at half maximum (FWHM) values of the beam were 2.2 and 2.3 μm in the *x* and *y* axes, respectively. With the TUT in the beam path, the FWHMs along the *x* and *y* axes were 2.32 ± 0.01 μm and 2.47 ± 0.05 μm, respectively (*SI Appendix*, Fig. S3*C*, *n* = 4). Since the differences in tightly focused beam profile with and without the TUT are at the submicrometer level, the beam quality with the TUT is well maintained.

Then, we compared the lateral and axial resolutions of the PAI system with those of the commercial UT (50 MHz) with the opto-ultrasound beam combiner, the ring UT, and the TUT (*SI Appendix*, Fig. S4 *A* and *B*). The lateral resolution of the TUT−PAI system is the best, thanks to its high optical NA. Notably, the optical NA in the ring−UT−PAI system is the lowest due to the limited hole size in the transducer center, resulting in relatively poor lateral resolution. However, the axial resolution of the TUT−PAI system is relatively low, owing to the narrow acoustic bandwidth. This issue will be further considered in *Discussion*. The PA SNRs of the three UTs were also compared (*SI Appendix*, Fig. S4*C*). The SNR of the TUT−PAI system is 12 dB higher than that of the ring−UT−PAI and 15 dB higher than that of the commercial−UT−PAI. Two factors largely account for the high SNR of the TUT−PAI: 1) Acoustic attenuation and reflection can be significantly minimized, a key issue for PAI with the commercial UT and opto-ultrasound combiner. 2) The tight acoustic focus of the TUT leads to smaller side-lobe signals, a key problem for the ring UT. All these parameters are quantified and compared in *SI Appendix*, Fig. S4*D*.

Next, we measured the sensitivity roll-off of the OCT system with and without the TUT (*SI Appendix*, Fig. S5). The input laser power on a sample was equalized in both measurements. Measured at a depth of 0.25 mm, the OCT sensitivities in both cases are identical, 38 dB, and the sensitivities roll off ∼17 dB at an imaging depth of 2.25 mm for both. This result shows that the TUT has little effect on the performance of the OCT system.

Finally, the FL characteristics with and without the TUT were analyzed (*SI Appendix*, Fig. S6). The FL sensitivity of fluorescein at 488 nm was measured at different concentrations, with and without the TUT. For the same laser output power with and without the TUT, the FL intensity was reduced by up to 53% with the TUT, due to two-way attenuation (red versus blue lines in *SI Appendix*, Fig. S6*A*). When using the TUT, we increased the laser output power by 58% to equalize the laser power incident on the sample (*SI Appendix*, Fig. S6*B*). The FL sensitivity with the TUT was reduced by up to 27% (only one-way attenuation for receiving FL signals) over the sensitivity without the TUT (red versus green lines in *SI Appendix*, Fig. S6*A*). This result agrees with the bidirectional light attenuation (50%) when 488-nm FL excitation and 510-nm FL emission light passes through the TUT. In particular, when using the TUT, the SNR was lower by 5.6 dB on average than without the TUT. After the input laser power was equalized, the difference in the average SNRs was only 2.2 dB (*SI Appendix*, Fig. S6*C*).

### In Vivo Monitoring of Rats’ Eyes after Chemical and Suture Injuries.

To investigate the in vivo capability of our quadruple fusion imaging system with the TUT, we monitored rats’ eyes after inflicting alkali burn (*n* = 5) and suture (*n* = 6) injuries. After such injuries, the eye exhibits one or more of the following changes related to serious vision loss, such as CNV, morphological changes, fibrous membrane and edema induced by inflammation, and cataracts. The comprehensive imaging provided by the quadruple fusion system is very well suited for observing this variety of changes.

**Fig. 4.**
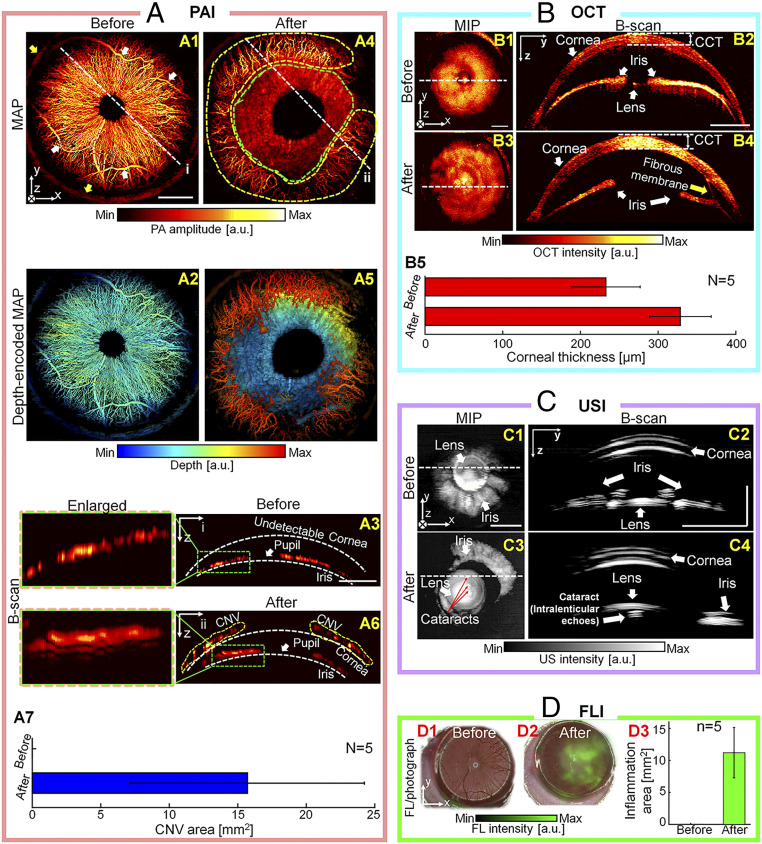
In vivo quadruple fusion imaging of rats’ eyes before and after alkali burns. (*A*) PA MAP, depth-encoded PA MAP, B-scan, and enlarged B-scan images before (*1*–*3*) and after (*4*–*6*) alkali burns, and the quantified CNV area (*7*). (*B*) OCT MIP and B-scan images before (*1* and *2*) and after (*3* and *4*) alkali burns, respectively, and the quantified CCT (5). (*C*) US MIP and B-scan images before (*1* and *2*) and after (*3* and *4*) the alkali burns, respectively. (*D*) Overlaid FL and photographic images of the inflammation areas before (*1*) and after (*2*) the alkali burns, and the quantified inflammation area (*3*). The rat cornea was stained with fluorescein. (Scale bar = 1 mm.) The error bars in *A*, *7*, *B*, *5*, and *D*, *3* indicate the SD.

**Fig. 5.**
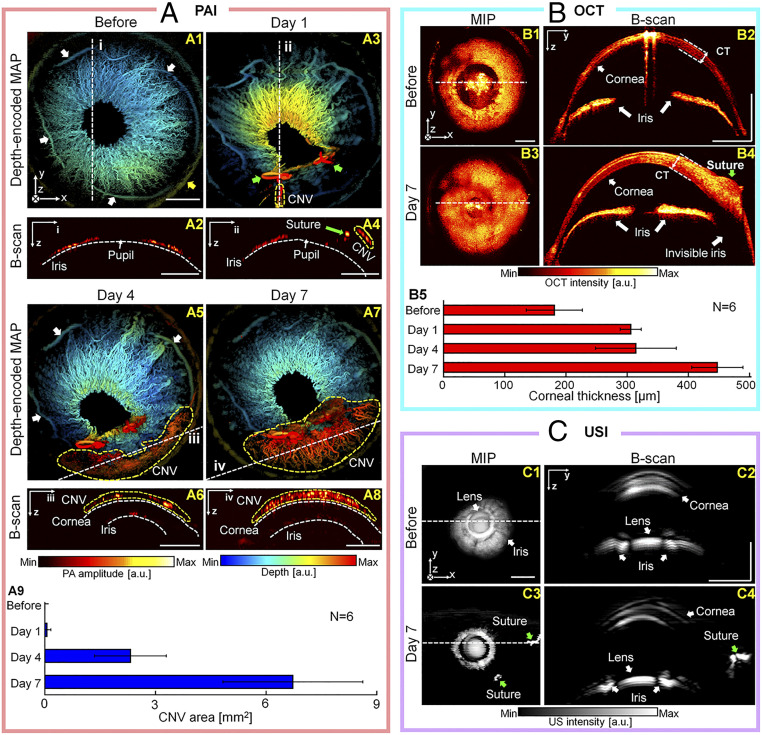
In vivo imaging of rat eyes before and after suture injuries, using the modalities of the quadruple fusion system. (*A*) Depth-encoded PA MAP and B-scan images acquired before (*1* and *2*) and on day 1 (*3* and *4*), day 4 (*5* and *6*), and day 7 (*7* and *8*) after suture injury, respectively, and the quantified CNV area (*9*). (*B*) OCT MIP and B-scan images before (*1* and *2*) and after (*3* and *4*) the suture injury, respectively, and the quantified CT (*5*). (*C*) US MIP and B-scan images before (*1* and *2*) and after (*3* and *4*) the suture injury, respectively. (Scale bar = 1 mm.) CT, corneal thickness.

**Fig. 6.**
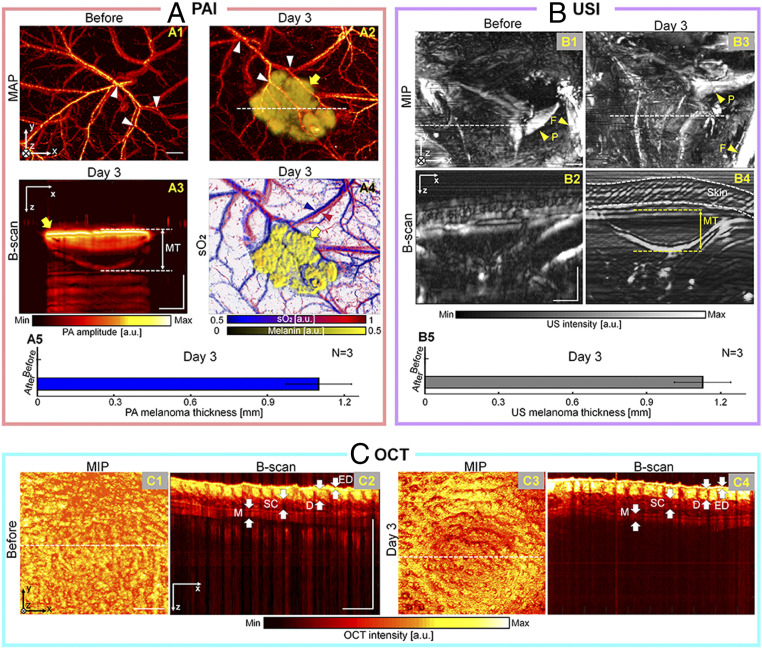
In vivo multimodal imaging of subcutaneous melanomas in a mouse model. (*A*) PA MAP images before (*1*) and on day 3 after (*2*) melanoma injection. The vascular network and spectrally unmixed melanoma are overlaid in *2*. PA B-scan image (*3*) on day 3 after the injection, acquired along the dashed line in *A2*. Overlaid PA sO_2_ and melanoma image (*4*). Quantification of the melanoma thickness based on the PA B-scan image (*5*). (*B*) US MIP and B-scan images before (*1* and *2*) and after (*3* and *4*) the melanoma injection. Quantification of the melanoma thickness based on the US B-scan image (*5*). (*C*) OCT MIP and B-scan images before (*1* and *2*) and after (*3* and *4*) the melanoma injection. (Scale bar = 1 mm.) ED, epidermis; D, dermis; SC, subcutis; and M, muscle.

**Fig. 7.**
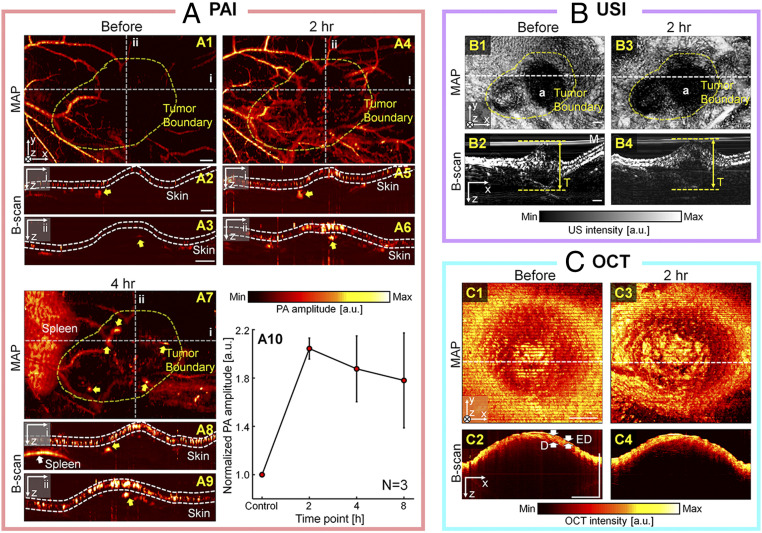
In vivo multimodal imaging of subcutaneous 4T1 breast carcinoma in a mouse model. (*A*) PA MAP and B-scan images before (*1* to *3*) and 2-h (*4* to *6*) and 4-h (*7* to *9*) after the PEG-GNRs labeling. Quantification of PA signal enhancements in tumor based on the PA MAP images (*10*). (*B*) US MIP and B-scan images before (*1* and *2*) and after (*3* and *4*) the PEG-GNRs labeling. (*C*) OCT MIP and B-scan images before (*1* and *2*) and after (*3* and *4*) the PEG-GNRs labeling. (Scale bar = 1 mm.) T, thickness.

Fig. 4 shows a rat’s eye before and after alkali burns, imaged with each of the four modalities in our system: PAI for monitoring CNV (Movie S1), OCT for imaging morphological changes and fibrous membrane (Movie S2), USI for imaging cataracts (Movie S3), and FLI for mapping inflammation. As seen in Fig. 4 *A*, *1*, before the injury, the root vessels of the iris (white arrows) and the limbal vessels (yellow arrows) are clearly visible in the PA maximum amplitude projection (MAP) image. In the depth-encoded PA MAP image processed by the Random Sample Consensus (RANSAC) algorithm (see *Materials and Methods* for details), a majority of the blood vessels are positioned in the iris at a relatively similar depth (Fig. 4 *A*, *2*). Further, no blood vessels are observed in the cornea above the iris, as shown in the PA B-scan image cut along line “i” (Fig. 4 *A*, *3*). One week after the alkali burn to the cornea surface and the limbal vessels, significant neovascularization has been induced in the cornea (yellow dashed region, Fig. 4 *A*, *4*). The precise depths where the neovascularization occurred are indicated in the depth-encoded PA MAP image (Fig. 4 *A*, *5*) and the B-scan image (cut along the line “ii”, yellow dashed regions, Fig. 4 *A*, *6*). Due to corneal opacity caused by the alkali burn, the iris vascular networks are not clearly delineated in the MAP image (green dashed region, Fig. 4 *A*, *4*), the depth-encoded MAP image (Fig. 4 *A*, *5*), or the B-scan image (Fig. 4 *A*, *6*). Flow-based OCTA can also clearly image eye blood vessels (*SI Appendix*, Fig. S7*A*). However, turbid media, such as a corneal opacity caused by alkali burn, can impair OCT imaging by disordering coherent light (*SI Appendix*, Fig. S7*B*) (55–57). Iris vessels under an opaque corneal injury are visible in PA images (cyan triangles in *SI Appendix*, Fig. S7 *B*, *3* and *4*), but not in OCTA images (*SI Appendix*, Fig. S7 *B*, *6* and *7*). Finally, the CNV areas are photoacoustically quantified to have an area 15.7 ± 7.6 mm^2^ at 1-wk postinjury (*n* = 5; Fig. 4 *A*, *7*). Second, the morphological changes in anterior segment were imaged by OCT (Fig. 4*B*) and USI (Fig. 4*C*). The OCT maximum intensity projection (MIP) image (Fig. 4 *B*, *1*), taken before the injury, shows the iris and lens. Note that the iris is flat and symmetrical around the lens as shown in the OCT B-scan image, and the central corneal thickness (CCT) is about 264 µm (Fig. 4 *B*, *2*). However, after the injury, the iris is no longer symmetrical (Fig. 4 *B*, *3* and *4*). In particular, the CCT has thickened to 356 µm, implying that corneal edema was induced by injury-associated inflammation. The average increase in CCT is 96 ± 31 µm (*n* = 5, Fig. 4 *B*, *5*). Interestingly, fibrotic membranous strand, which is induced by severe inflammation, was observed (yellow arrow) in the OCT image (45). Furthermore, before the alkali burn, the normal iris and lens are also clearly discernable in the US MIP and B-scan images (Fig. 4 *C*, *1* and *2*). After the injury, the iris structures are changed, and cataracts appear in the lens (three white dots indicated by red arrows) (Fig. 4 *C*, *3* and *4*). Cataract is another serious complication of chemical burns, and causes vision loss (41, 42). Finally, the corneal epithelial inflammation causing the corneal edema is fluorescently confirmed with fluorescein stain. Overlaid FL and photographic images taken before and after the chemical injury are shown in Fig. 4 *D*, *1* and *2*. Based on the FL image, the quantified inflammation region measures 11.2 ± 3.5 mm^2^ (Fig. 4 *D*, *3*). Histological evaluations with hematoxylin and eosin (H&E) staining and CD31 immunostaining were performed on ocular tissue extracted 7 d after alkali burns (*SI Appendix*, Fig. S8).

As the second ophthalmic application, we used the quadruple fusion imaging system to monitor the consequences of suture injury to the rats’ eyes. It is well known that suture injury can induce angiogenesis and associated complications (e.g., edema), but the structural damages to the cornea are relatively less compared to those resulting from chemical injury. Fig. 5 shows quadruple fusion images of a rat’s eye before a suture injury and on day 1, day 4, and day 7 after the injury. PAI for was used for monitoring CNV, and OCT was used for monitoring morphological changes in an anterior segment. Before the suture injury, the iris vasculature, including the root iris (white arrows) and limbal vessels (yellow arrows), is clearly visualized in the depth-encoded PA MAP image (Fig. 5 *A*, *1*). In the PA B-scan image cut along the line “I,” the iris blood vessel networks are symmetrical around the pupil (Fig. 5 *A*, *2*). In the depth-encoded PA MAP image captured at 1 d postinjury, the sutures are distinguishable (green arrows in Fig. 5 *A*, *3* and *4*), and early-stage CNV is visible (yellow dashed regions in Fig. 5 *A*, *3* and *4*). At 4 d postinjury, neovascularization is prominent from the limb vessels to the sutures (yellow dashed regions in Fig. 5 *A*, *5* and *6*) (39). The corneal and iris blood vessels are also clearly identifiable in the B-scan cut along the line “iii” (Fig. 5 *A*, *6*). Vascular growth and CNV have progressed by day 7 (yellow dashed regions in Fig. 5 *A*, *7* and *8*). The iris blood vessels under the CNV area are faint, but recognizable, in the B-scan image cut along the line “iv” (Fig. 5 *A*, *8*). The CNV areas on postsurgery days were photoacoustically analyzed, and the CNV was found to cover an area of 6.7±1.7 mm^2^ on day 7 (Fig. 5 *A*, *9*). Further structural complications, such as surgery-associated edema, were monitored by OCT MIP imaging (Fig. 5*B*). OCT B-scan images (Fig. 5 *B*, *2* and *4*) obtained before the suture surgery and on day 7 afterward (Fig. 5 *B*, *1* and *3*) show that the iris symmetry is well maintained after surgery, confirming relatively modest damage to the anterior segment. The corneal thickness was 198 µm before the suture injury, but had thickened to 460 µm on day 7. In all the suture injury cases, the corneal thickness increased by an average of 260 ± 62 µm during the postinjury time period, suggesting that edema occurs in the cornea (Fig. 5 *B*, *5*; *n* = 6). In the US B-scan image (Fig. 5 *C*, *2*, cut along the line in Fig. 5 *C*, *1*) of the “before suture injury,” the lens is clearly delineated in the center of the iris. On day 7 after the suture injury, two sutures are identifiable in the US MIP and B-scan image (Fig. 5 *C*, *3* and *4*, green arrows). Overall, as shown in Fig. 5*C*, there is apparently no significant difference in images of the anterior segment before and after the suture injury, and thus it can be inferred that the injury does not significantly affect the eye’s structure. Histological evaluations with H&E staining and CD31 immunostaining were performed on ocular tissue extracted 7 d after suture injuries (*SI Appendix*, Fig. S8).

### In Vivo Multimodal Imaging of Pigmented (Melanoma) and Nonpigmented (4T1 Breast Carcinoma) Tumors.

To further explore the capability of our multimodal imaging system, we imaged subcutaneous melanomas (*n* = 3, pigmented) and 4T1 tumors (*n* = 3, nonpigmented) in a mouse model. For these oncologic applications, we used a near-infrared (NIR) tunable laser for PAI to image deeper regions than possible with visible wavelengths. Fig. 6 shows label-free triple fusion images of a mouse’s body before and after subcutaneous melanoma injection. Before the melanoma injection, the background vascular network is well delineated in the PA MAP image at 532 nm (Fig. 6 *A*, *1*). At 3 d postinjection, the melanoma and surrounding blood vessels are clearly visualized (Fig. 6 *A*, *2*, yellow arrow for melanoma). Because melanoma itself contains pigment, strong PA signals can be acquired from it without any external contrast agent. The melanoma was spectrally unmixed by using five wavelengths (730, 756, 778, 796, and 818 nm) of the NIR light source (*SI Appendix*, *Methods S2*). The white triangles represent the locations of the same blood vessels in the preinjection and postinjection PA MAP images (Fig. 6 *A*, *1* and *2*). The thickness of the melanoma (1 mm), the key parameter determining the melanoma stage, is clearly indicated in the PA B-scan image (Fig. 6 *A*, *3*), and matches well with the histological result (890 μm; *SI Appendix*, Fig. S9). Moreover, we computed the relative sO_2_ map for the surrounding arteries and veins (Fig. 6 *A*, *4*). The averaged maximum PA thickness is 1.1 ± 0.1 mm (*n* = 3 and Fig. 6 *A*, *5*). Further, tumor-associated morphologies were ultrasonically imaged before the melanoma injection and on day 3 after the injection. In the US MIP images, where the skin signals are removed, bone structures, including a femur and pelvis, are identified (Fig. 6 *B*, *1* and *3*). From the US B-scan images (Fig. 6 *B*, *2* and *4*) cut along the white dashed lines in the US MIP images (Fig. 6 *B*, *1* and *3*), respectively, only the US B-scan image (Fig. 6 *B*, *4*) acquired at 3 d postinjection shows the hypoechoic mass presumed to be the melanoma. The averaged maximum melanoma thickness is 1.1 ± 0.1 mm (*n* = 3 and Fig. 6 *B*, *5*), which matches well with the PA result (Fig. 6 *A*, *5*). Detailed skin layers such as the epidermis, dermis, subcutis, and muscle are depicted by OCT. In the OCT B-scan images (Fig. 6 *C*, *2* and *4*) cut along the white dashed lines in the OCT MIP images (Fig. 6 *C*, *1* and *3*), respectively, no significant changes are observed in the skin layer before and after the melanoma injection. The presence of the melanoma under the skin and its thickness are confirmed by the H&E staining histopathology image (*SI Appendix*, Fig. S9).

As a second oncologic application, we monitored the passive uptake of PEGylated gold nanorods (PEG-GNRs; *SI Appendix*, *MethodsS3*) in 4T1 tumor-bearing mice over time, at 2, 4, and 8 h postinjection. Fig. 7 shows a label-free image as a control, along with PEG-GNRs-labeled 4T1 tumor images of a mouse 7 d after subcutaneous injection of the 4T1 tumor cells. In Fig. 7*A*, the same dynamic range was applied to all PA images to intuitively confirm the PA signal enhancement by the contrast agent. Further, to confirm the tumor boundary, a USI-guided tumor boundary (yellow dashed lines) was added to the PA MAP images. Before the intravenous PEG-GNRs labeling, mainly the surrounding blood vessels outside of the tumor region are imaged (Fig. 7 *A*, *1*). In the PA B-scan images cut along lines “i” and “ii” of Fig. 7 *A*, *1*, weak PA signals are indicated by the yellow arrows (Fig. 7 *A*, *2* and *3*). At 2 h postinjection of the PEG-GNRs, distinct PA signal enhancement is captured in the tumor boundary region of the PA MAP image (Fig. 7 *A*, *4*, yellow dashed region). In particular, the blood vessels become clearly visible on the right side of the image, suggesting that the PEG-GNRs are circulating. In the PA B-scan images (Fig. 7 *A*, *5* and *6*) cut along the lines “i” and “ii” of Fig. 7 *A*, *4*, enhanced PA signal localization is markedly visualized, as indicated by the yellow arrows, which are at the same locations as in the prelabeling images. In the PA MAP image at 4 h postlabeling, the PA signals within the tumor boundary further increase (yellow arrows in Fig. 7 *A*, *7*). However, the PA signals from the blood vessels decrease, possibly because the PEG-GNRs may have accumulated in the skin layer and absorbed the light from the skin surface. This possibility can be confirmed by the strong PA signals from the skin layer shown in the B-scan images (Fig. 7 *A*, *8* and *9*, cut along lines “i” and “ii” of Fig. 7 *A*, *7*). In these B-scan images, PA signal enhancement is also seen in the same location indicated by the yellow arrows in the prelabeling B-scan images. In particular, after 4 h postlabeling, strong PA signals are observed in the spleen (Fig. 7 *A*, *7* and *8*). Based on the PA MAP images and tumor boundaries (*n* = 3), the quantified PA signal enhancements were, respectively, 104 ± 9%, 88 ± 27%, and 76 ± 40% at 2, 4, and 8 h postinjection. Next, USI was performed to evaluate the tumor area. In USI, a dense or solid tissue such as a tumor is hypoechoic. Using this feature, the tumor boundaries were delineated in the prelabeling and postlabeling US MIP images (Fig. 7 *B*, *1* and *3*), and applied to the PA quantification. In the B-scan images cut along the white dashed lines of the US MIP images, the thickness of the tumor was measured with reference to the lower boundary of the tumor and the upper boundary of the skin (Fig. 7 *B*, *2* and *4*). The evaluated tumor thicknesses at prelabeling and 2 h postlabeling are 5.0 and 4.9 mm, respectively. For detailed skin layer observation, OCT imaging was performed in region “a” of the US MIP images. In the prelabeling and the postlabeling OCT B-scan images (Fig. 7 *C*, *2* and *4*) cut along the white dashed lines of OCT MIP images (Fig. 7 *C*, *1* and *3*), the epidermis and dermis layers are clearly visualized, with no significant changes. The presence of the 4T1 tumor under the skin and its thickness are confirmed by an H&E stained histopathology image (*SI Appendix*, Fig. S10). The accumulation of the PEG-GNRs in the 4T1 tumor and skin at 8 h postinjection is confirmed by the inductively coupled plasma mass spectrometry (ICP-MS) analysis (*n* = 3; *SI Appendix*, Fig. S11).

## Discussion

Many researchers have pursued the integration of US and OI systems to improve the sensitivity, accuracy, and resolution of the resultant images. However, in these efforts, the opacity of the UT dictated noncoaxial sensor paths. The implementations typically employed a beam combiner or coupler to bypass the light or to tilt either the light or US to overlap the transmit and receive signals. These modifications made the images suboptimal, added bulk to the system, and limited the possible applications. The quadruple fusion imaging system that we present here seamlessly integrates USI, PAI, OCT, and FLI, enabled by employing a TUT that maximizes the SNR and minimizes the complexity of the system. Our spherically focused TUT has a 9-mm element size, dual-center frequencies of 7.5 MHz and 31.5 MHz, and higher than 70% transparency in the visible and NIR optical wavelength ranges.

To validate the performance of our quadruple fusion imaging system, the system was compared to conventional individual USI, PAI, OCT, and FLI systems. First, the TUT itself showed a higher US transmission SNR (≥11 dB) and a tighter focal point (≥1.8-fold) compared to commercial UTs and customized ring UT. Second, the TUT−PAI showed higher lateral resolution than conventional− and ring−UT−PAIs, thanks to its high optical NA. However, the narrow bandwidth of the TUT made the axial resolution of the TUT−PAI relatively low. In addition, the TUT−PAI showed an SNR more than 12 dB higher than those of the conventional− and ring−UT−PAIs, thanks to the seamless coaxial alignment of the light and US. Third, the sensitivity roll-offs of the OCT system with and without the TUT were identical (17 dB) at an imaging depth of 2.25 mm. Lastly, the SNR of the TUT−FLI was lower by 2.2 dB, mainly caused by bidirectional attenuation during FL excitation and emission. All these results suggest that the imaging capabilities of our quadruple fusion system are comparable to those of conventional individual USI, PAI, OCT, and FLI systems.

As the first biological application, we successfully used the quadruple fusion imaging system to monitor the in vivo responses of rats’ eyes to injuries (*n* = 5 for alkali burns and *n* = 6 for suture injuries). Both injuries mainly cause CNV, inflammation, and edema, which can lead to blindness (58). In particular, chemical burns can cause serious complications (e.g., cataracts and severe anterior chamber reaction). To begin, we effectively monitored and quantified the progression of the CNV through depth-resolved PAI and OCTA. Then, in OCT images, we morphologically and quantitatively confirmed that the cornea became thicker after both injuries, implying edema formation. Further, fibrous membrane induced by anterior chamber reaction was also visualized in the OCT image. Next, after inflicting alkali burns, we used USI to observe changes in the anterior segment and cataracts in the lens. Finally, for the alkali burn injuries, corneal epithelial inflammation was quantified by fluorescein staining FLI. These results prove that our quadruple ophthalmic imaging system based on the TUT can provide complementary information about individual USI, PAI, OCT/OCTA, and FLI results. Although we focused on monitoring anterior segment diseases in this study, our imaging system can serve as a comprehensive ophthalmic imaging and diagnostic tool, including imaging posterior segment diseases (e.g., floating layer, macular degeneration, and diabetic eye).

As a second application, we imaged pigmented (*n* = 3, melanoma) and nonpigmented (*n* = 3, 4T1 breast carcinoma) tumor-bearing mice in vivo to demonstrate label-free and labeled cancer monitoring, respectively. In the melanoma mice, we used PAI alone to measure the thickness of the melanoma, without any exogenous agent. Further, the melanoma thickness and sO_2_ map were calculated from the multiwavelength PA images by using spectral unmixing. In the 4T1 carcinoma mice, we monitored and quantified the PA signal enhancement over time after PEG-GNRs labeling. Using USI, the hypoechoic properties of tumors were exploited to measure the tumor thicknesses and lateral boundaries for US-guided PA quantification. Further, in the melanoma mice, bone structures such as the pelvis and the femur were ultrasonically observed near the melanoma, but no differences were found before and after the melanoma injection. In OCT images, we delineated detailed skin layers in both tumor models, and there was no visible difference in images captured before and after injection of cancer cells or prelabeling and postlabeling with PEG-GNRs. Unfortunately, despite the early stage of the tumors, the tumor thicknesses exceeded the distinguishable depth for OCT imaging, so it was not possible to identify the lower tumor boundaries. These results demonstrate that our multimodal imaging system can serve as a monitoring tool for pigmented and nonpigmented tumors. Furthermore, we believe that, by providing comprehensive monitoring results, our system can aid robust diagnosis of various skin diseases (e.g., basal cell carcinoma, squamous cell carcinoma, psoriasis, and scleroderma).

A multimodal imaging system has potential for many other clinical applications that can benefit from its enhanced sensitivity/specificity in providing better interventional outcomes (50, 51), for example, needle guidance, lymph node identification, and interventional cardiology. Perhaps most notably, the integrated system could be miniaturized for intravascular imaging, with potential for clinical translation. Intravascular US (IVUS), intravascular OCT (IV-OCT), intravascular PA (IVPA), and/or intravascular NIR spectroscopy (IV-NIRS) or fluorescence (IV-NIRF) have been actively investigated. Combinations of these modalities, such as dual modes (e.g., IVUS−OCT, IVUS−PA, and IVUS−NIRS(F)) or triple modes (e.g., IVUS−OCT−PA, IVUS−OCT−NIRS(F), and IVUS−PA−NIRS(F)) hold great promise for imaging coronary artery diseases (14, 15, 23, 52, 53). The integration of all four modalities into one system can provide a full range of physiological information associated with coronary artery disease, such as the intervessel morphology, lipid formation, thickness of the thin fibrous cap, and inflammation.

While we successfully demonstrated the integrated imaging system with the TUT, we faced many challenges, including fabricating the TUT and minimizing acoustic impedance mismatch between the materials. These challenges resulted in a relatively narrow frequency band, yielding low axial resolution. This problem could be overcome by identifying an acceptably transparent material with a high acoustic impedance to fabricate the BL. Also, fabricating a one−three or two−two piezoelectric composite with a transparent epoxy could lower the acoustic impedance of the piezoelectric layer so that no matching is required. In the future, we will explore materials for optimal impedance matching, and conduct parametric studies on various sizes and frequency bands of TUTs. Additionally, our spectral domain OCT (SD-OCT) system can be updated to provide deep structural information and angiography. A swept-source OCT (SS-OCT), which uses relatively long wavelengths, is probably a good alternative. The quadruple fusion imaging system with SS-OCT will be further studied in the future.

## Materials and Methods

### Optical Transmittance Measurement.

We used an ultraviolet−visible−NIR spectrophotometer (Cary 60 UV-Vis, Agilent) to measure the optical transparencies of the AgNWs only, the LNO only, and the TUT in the range between 200 and 900 nm.

### Customized Ring and Commercial UTs.

A customized single-element ring UT was made using a 36° rotated Y-cut LNO single crystal (Boston Piezo-Optics, Inc.), and fabricated to have a central frequency of 20 MHz. The ring UT was 9 mm thick and 10 mm in diameter, with a 2-mm hole in the center through which the laser beam could pass. The focal length of the ring UT was 20 mm. As representative commercial UTs, we selected two lens-focused designs with center frequencies of 20 MHz (V372-SU, Olympus NDT) and 30 MHz (V375-SU, Olympus NDT) to use. The diameters and focal lengths of the commercial UTs were 6 and 19 mm, respectively.

### Acoustic Pressure Field Measurement.

The acoustic pressures of the TUT, ring UT, and commercial UTs were experimentally measured in degassed and deionized water under linear propagation conditions by using a hydrophone (HGL-0085, ONDA Corp.) and an acoustic intensity measurement system (AIM III, ONDA Corp.). The hydrophone was precisely aligned at the US focal point of each UT. Acoustic pressure fields were obtained by scanning the hydrophone 6 mm in the *x* axis and 6 mm in the *z* axis.

### Silver Nanowires as Transparent Electrodes.

We used AgNWs (*SI Appendix*, Fig. S12) as transparent electrodes to operate the TUT. The AgNWs were dispersed in ethanol with 0.5 wt% (Nanopyxis Co., Ltd). Further, the AgNWs solution was diluted 10 times with ethanol. Using an N_2_ pressure of 0.24 MPa, 2 mL of the AgNW solution was spray-coated on the LNO. During the spray coating, the LNO was maintained at 90 °C to remove residual solvent. The sheet resistance of the spray-coated AgNWs was 9.7 Ω sq^−1^.

### Seamlessly Integrated Quadruple Fusion Imaging System Using the TUT.

A schematic diagram of the quadruple fusion imaging system integrating USI, PAI, OCT, and FLI is shown in Fig. 3.

For PAI, two types of pulsed laser source are used for excitation: 1) a Nd:YAG laser (SPOT-532, Elforlight; wavelength: 532 nm; pulse duration: 1.8 ns) and 2) a Ti:Sapphire tunable laser (AWAVE-Ti:S-700-900, Advanced Optowave Corp.; wavelength: 700 to 900 nm, linewidth: <3 nm). The collimated beam from the Nd:YAG laser, after passing through a DM (DM1, DMLP505R, Thorlabs), is coupled to a single mode fiber (SMF1, P1-460B-FC, Thorlabs). The collimated beam from the Ti:Sapphire laser is coupled to an MMF with a 50-μm core size (MMF, M16L01, Thorlabs). PA excitation can be switched easily by flipping the mirror and changing the input fiber. The optical beam exiting the fiber is further collimated to a 6-mm diameter by a CL (AC254-030-A, Thorlabs). DM2 (FF505-SDi01, Semrock) is removed when performing PAI. The collimated 532-nm laser beam passing through DM3 (#69-218, Edmund Optics) is focused on the sample through an OL (OL1, AC254-030-AB, Thorlabs). Note that DM3 is temporarily removed when using the Ti:Sapphire laser. A custom-made water tank is placed on top of the sample for acoustic coupling, and the lower surface of the TUT is immersed in the water. The *x*–*y* motorized stages (L-509, Physik Instrumente) connected to the imaging head are used for raster scanning. The PA signals are amplified through a P/R (5072PR, Olympus NDT), digitized by 200 MS/s sampling via a digitizer board (PCI-5124, National Instruments), and saved on a PC. The motorized stages and P/R are synchronized, performed by trigger signals from a multifunction electronic board (PCIe-6320, National Instruments). The fields of view (FOVs) of the PA images measure 5 mm × 5 mm × 3 mm (*x*, *y*, and *z* axes) in a rat’s eye and 15 mm × 10 mm × 3.8 mm in a mouse tumor. The data acquisition times for 3D PA data are 9 min 17 s for the eye and 18 min 6 s for the cancer, respectively (*SI Appendix*, Table S1).

For USI, we used the P/R to transmit, receive, and internally amplify the US signals. The TUT was driven by the P/R with a negative impulse of 180 V and damping of 50 Ω. A 50-MHz low-pass filter between the TUT and P/R eliminated the superimposed high-frequency noise. The mechanical raster scanning and data acquisition methods were the same as for PAI. The FOV of the 3D US image was 5 mm × 5 mm × 3.8 mm in a rat’s eye and 15 mm × 10 mm × 7.7 mm in a mouse tumor. The lateral and axial resolutions of the USI system, measured by imaging a carbon fiber phantom (*SI Appendix*, Fig. S13), were 102 and 81 μm, respectively. The data acquisition time was the same as for PAI (*SI Appendix*, Table S1).

For OCT and OCTA, we implemented an SD-OCT system (59). An NIR CW SLED (SLD-351-HP3, Superlum) with a central wavelength of 860 nm and a bandwidth of 50 nm was used as the SD-OCT light source. The light from the SLED passed through a 50:50 fiber optic coupler (FC850-40-10-APC, Thorlabs), and the two fibers were separately connected to a sample arm and a reference arm. The spectrometer for SD-OCT consisted of a collimator (F280APC-B, Thorlabs), a transmission type diffraction grating (WP-HD1800/840, Wasatch Photonics), and a 12-bit 2,048-pixel line scan CMOS camera (SPL2048-140K, Balser). A frame grabber (PCIe-1429, National Instruments) captured the interference OCT signals from the sample and reference arms. The OCT beam was aligned coaxially with the TUT passing through the GMs (GVS002, Thorlabs), DM3, and OL1. Two GMs were used for OCT beam scanning on the *x*–*y* axes. The DM3 reflected the beam 90°, and the reflected beam was focused through OL1 to illuminate the sample. The FOVs of a rat eye and mouse tumor were 6 mm × 6 mm × 3 mm and 4.8 mm × 4.8 mm × 3.4 mm, respectively. To collect the dataset for OCTA, the system was operated at a B-frame rate of 60 Hz. Each B-scan, consisting of 750 A-line scans, was acquired 10 times at the same position. To reconstruct the OCTA, we employed an eigen decomposition-based OCTA algorithm for the data (60). In the ophthalmologic application, for 3D OCT and OCTA data, acquisition times were, respectively, 40 s and 2 min 20 s. In the oncologic application, it took 32 s to acquire 3D OCT data (*SI Appendix*, Table S1).

For FLI, we implemented a 2D planar CMOS camera-based system. A 488-nm-wavelength CW laser source (Cobolt 06-MLD, Cobolt Laser) excited a 0.1% fluorescein dye (Fluorescein sodium salt, Sigma-Aldrich) emitting the FL signals. The collimated laser output was reflected by a mirror and then coupled to the same SMF1 used for PAI through DM1. The laser beam from the fiber was passed through DM2, DM3, OL1, and TUT coaxially and focused on the sample. After the emitted FL light was reflected 90° by DM2, the desired light was passed through an optical filter (OF, #86-951, Edmund Optics) and collected by the CMOS camera (HK3.1, KOPTIC). The FOV of the 2D FL image from rat eye imaging was 6 mm × 6 mm on the *x* and *y* axes, respectively. The 2D FL image acquisition was done in real time (*SI Appendix*, Table S1).

### Automatic Ocular Surface Measuring Algorithm.

To automatically quantify the CNV, we used RANSAC based on an ocular surface detection algorithm (61).

### Cell Culture.

B16-F1 and 4T1 cell lines were purchased from American Type Culture Collection. B16-F1 and 4T1 cells were respectively cultured in Dulbecco’s modified Eagle’s medium (Gibco) and RPMI medium (HyClone) containing 9% fetal bovine serum (Gibco) and 100 U/mL penicillin/streptomycin (Gibco). The cells were incubated in humidified incubators maintained at 37 °C and 5% CO_2_.

### Animal Preparation.

All animal experimental procedures were conducted according to laboratory animal protocols approved by the Institutional Animal Care and Use Committee of the Pohang University of Science and Technology, and the regulations of the National Institutes of Health *Guide for the Care and Use of Laboratory Animals* (62). We prepared normal Sprague-Dawley rats (6 wk, 200 g) for the experiments. The rats were initially anesthetized with 4% isoflurane vaporized by inhalation gas (1.0 L/min flow rate) and kept under anesthesia with 2% isoflurane during the imaging. The anesthetized rat was placed on a heating pad on top of the manual stage to maintain a body temperature of 36.5 °C. As an acoustic impedance matching material, US gel was spread on the surface of the ROI to acquire the US and PA images. Alkali burn and corneal suture injuries were performed on the left eyes of the rats. To regulate the alkali burn injury area, we used 1.5-mm-diameter disks of Whatman filter paper that were saturated with 1 N NaOH solution. Under anesthesia with 3.5% isoflurane, the saturated piece of paper was placed on the rat’s eye for 30 s, and then the eye was rinsed with sterile saline solution. For corneal suture surgeries, the rats were first anesthetized via intraperitoneal injection with 0.2 mL of a mixture of ketamine (9 mg/kg) and xylazine (1 mg/kg). The cornea was artificially perforated with 10.0 nylon suture, placed eccentrically in the corneal stroma about 1.5 mm from the limbus. In FLI experiments, fluorescein staining was performed by gently dropping the solution onto the corneal surface, using 2 μL of 0.1% fluorescein dye solution dissolved in phosphate-buffered saline. In oncologic studies, Balb/c nude female mice (6 wk, 16 g) were used. The mice were inoculated subcutaneously in the left flank with 7 × 10^5^ B16-F1 cells for melanoma cancer and 7 × 10^5^ 4T1 cells for breast carcinoma. After 3 d for melanomas and 7 d for 4T1 tumors, the mice were anesthetized by 1.5% isoflurane, and the cancers were monitored. In the case of 4T1 tumor monitoring, the mice were intravenously injected with 200 μL of PEG-GNRs (11.9 nM), and the experiments were performed at 2, 4, and 8 h postinjection.

### Quantification of PEG-GNR Accumulation in Tumor by ICP-MS.

After monitoring 4T1 tumors for 8 h, the mice were euthanized with CO_2_, and the tumors and skins were excised, sequentially. To quantify the PEG-GNR accumulated in the tumors and skins, the amount of Au in the tumors and skins was measured. The tumors and skins were treated with aqua regia, and the temperature was elevated to 80 °C to digest organic molecules completely. The Au amount in the tumors and skins was quantified using ICP-MS (NexION-300D, PerkinElmer). A calibration curve was obtained with a commercially available gold standard (TraceCERT, Sigma Aldrich). The amount of Au was calculated as the percent of injected dose per weight of tumor.

## Supplementary Material

Supplementary File

Supplementary File

Supplementary File

Supplementary File

## Data Availability

All study data are included in the article and *SI Appendix*.
