# Supplementary material for: Quadruple ultrasound, photoacoustic, optical coherence, and fluorescence fusion imaging with a transparent ultrasound transducer
Source: Proc Natl Acad Sci U S A. 2021 Mar 8;118(11):e1920879118. doi: 10.1073/pnas.1920879118 (PMC7980418; doi:10.1073/pnas.1920879118)
Supplement: Supplementary File [file pnas.1920879118.sapp.pdf]

PNAS
www.pnas.org

Supplementary Information for

Quadruple Ultrasound, Photoacoustic, Optical Coherence, and Fluorescence Fusion Imaging with a Transparent Ultrasound Transducer

Jeongwoo Park[1,§], Byullee Park[1,§], Tae Yeong Kim[1], Sungjin Jung[1], Woo June Choi[2], Joongho Ahn[1], Dong Hee Yoon[3], Jeongho Kim[3], Seungwan Jeon[1], Donghyun Lee[1], Uijung Yong[1], Jinah Jang[1], Won Jong Kim[1], Hong Kyun Kim[3,*], Unyong Jeong[1,*], Hyung Ham Kim[1,*], and Chulhong Kim[1,*]

§These authors contributed equally to this work

[1]Department of Electrical Engineering, Creative IT Engineering, Materials Science and Engineering, Chemistry, Mechanical Engineering, School of Interdisciplinary Bioscience and Bioengineering, and Medical Device Innovation Center, Pohang University of Science and Technology, 77, Cheongam-Ro, Nam-Gu, Pohang 37673, Republic of Korea
[2]School of Electrical and Electronics Engineering, Chung-Ang University, 84 Heukseok-Ro, Dongjak-Gu, Seoul 06974, Republic of Korea
[3]Department of Ophthalmology, School of Medicine, Kyungpook National University, 680 Gukchaebosang-Ro, Jung-Gu, Daegu 41944, Republic of Korea

Corresponding authors: *Chulhong Kim, Hyung Ham Kim, Unyong Jeong, Hong Kyun Kim.

Email: chulhong@postech.edu, david.kim@postech.ac.kr, ujeong@postech.ac.kr, okeye@knu.ac.kr

This PDF file includes:

Supplementary text
Figures S1 to S14
Table S1
SI Reference

Other supplementary materials for this manuscript include the following:

Movies S1 to S3

Supplementary Information Text

Methods

Method S1. Krimholtz-Leedom-Matthaei model simulation using PiezoCAD.

The Krimholtz-Leedom-Matthaei (KLM) model, one of the widely used one-dimensional models for designing a piezoelectric single crystal ultrasound transducer, allows researchers to better understand and optimize the transducer's acoustic and electrical performance. Together with the KLM model, we used the PiezoCAD (Sonic Concepts, Inc., USA) simulation tool, which is the most commonly used software for transducer design. The properties of each material in the design, such as its electromechanical coupling coefficient, longitudinal velocity, density, and attenuation, were all first set to default values. The physical dimensions of the elements, such as their shape, size, and thickness, were then set as parameters. For the individual layers of the TUT, we then chose LNO as a piezoelectric material, non-conductive epoxy as a backing layer, N-SF11 plano-concave lens as an acoustic lens and also a first matching layer, and parylene as a second matching layer. We initially set the thickness of the LNO to half lambda, based on the desired center frequency of the transparent transducer. The thickness of each matching layer was set to $1/4^{th}$ lambda, corresponding to the center frequency. Iterative simulation was then carried out using the thickness of each layer and the diameter of the transducer as parameters to find the optimal value for the desired dual frequencies of 8 MHz and 30 MHz. All the simulations were conducted assuming a water medium.

Method S2. Spectral Unmixing Algorithm.

Spectral unmixing of each PA B-scan image was performed as follows[1,2]: 1) To compensate for the attenuation of the optical fluence as the tissue depth increased, the background signal was calculated for each depth, and then the fluence was normalized by using these calculated values. 2) The fluence-compensated multispectral PA images were spectrally unmixed to the components of the oxy-hemoglobin (HbO_2), the deoxy-hemoglobin (HbR), and the melanin by using the pseudo-inverse matrix approach:

$$[C_1 C_2 \cdots C_n] = [PA_1 PA_2 \cdots PA_k] \cdot M^T \cdot [M \cdot M^T]^{-1},$$

$$M = \begin{bmatrix} \mu_{\lambda_1}^1 & \mu_{\lambda_2}^1 & \cdots & \mu_{\lambda_k}^1 \\ \mu_{\lambda_1}^2 & \mu_{\lambda_2}^2 & & \mu_{\lambda_k}^2 \\ \vdots & & \ddots & \vdots \\ \mu_{\lambda_1}^n & \mu_{\lambda_2}^n & \cdots & \mu_{\lambda_k}^n \end{bmatrix}.$$

where C_n is the concentration of the nth component, PA_k is the fluence-compensated PA image at the kth wavelength, and $\mu_{\lambda_k}^n$ is the normalized optical absorption coefficient of the nth component at the kth wavelength. By using these unmixed components of the HbO_2 and HbR, the relative sO_2 map was computed as follows:

$$sO_2 = \frac{HbO_2}{HbO_2 + HbR}$$

Method S3. Gold Nanorod Synthesis and PEGylation of Gold Nanorods

We purchased cetyltrimethylammonium bromide (CTAB), gold(III) chloride hydrate ($HAuCl_4$), ascorbic acid, and silver nitrate ($AgNO_3$) from Sigma-Aldrich (USA). Sodium borohydride ($NaBH_4$) was supplied from Honeywell Fluka (USA). mPEG-SH (2kDa) was purchased from SunBio (Republic of Korea). All reagents were used as received without further purification. The GNRs were prepared with a seeded-growth mechanism previously described[3,4]. Gold seeds were prepared by adding 0.6 mL of 0.01M pre-chilled $NaBH_4$ solution to a 5 mL mixed solution containing 0.1 M CTAB and 0.25 mM $HAuCl_4$. Then the mixture was shaken for 2 minutes with vigorous stirring. The growth solution was prepared by mixing 125 mL of 0.2 M CTAB, 125 mL of 1 mM $HAuCl_4$, and 8 mL of 4 mM $AgNO_3$. This orange-colored solution became transparent after adding 1.75 mL of 78.8 mM ascorbic acid for Au ion reduction. Finally, 0.3 mL of a brown-colored seed solution was added to the transparent growth solution, and the mixture became purple/brownish over 60 minutes. After the reaction, the GNR solution was purified by centrifugation at 16,000 rcf for 20 minutes. The purification step was repeated twice more to eliminate the remaining CTAB. The molar extinction coefficient, calculated from previously published literature[5], was 3.46×10^9 M^{-1} cm^{-1}.

PEGylation of the GNRs was conducted by adding mPEG-SH (2kDa) to exchange the CTAB capped at the surface of the GNRs. A solution of mPEG-SH in distilled water (DW) was added to the GNRs in DW, and then the solution was stirred for 12 hr to obtain PEG-GNRs. After the reaction, the PEG-GNRs were purified by centrifugation at 16,000 rcf for 20 minutes. The characteristics of the PEG-GNRs, including their optical absorption properties, zeta potential, and transmission electron microscope (TEM) images, are summarized in *SI Appendix*, Fig. S14. The zeta potential was measured using a Nano Z (Malvern Instruments, UK). High-resolution TEM images with electron energy loss spectroscopy elemental mapping were obtained using a JEM-2200FS electron microscope (JEOL, Japan). All UV-Vis absorption spectra were obtained using a SpectraMax i3 plate reader (Molecular Devices, USA).

Figures

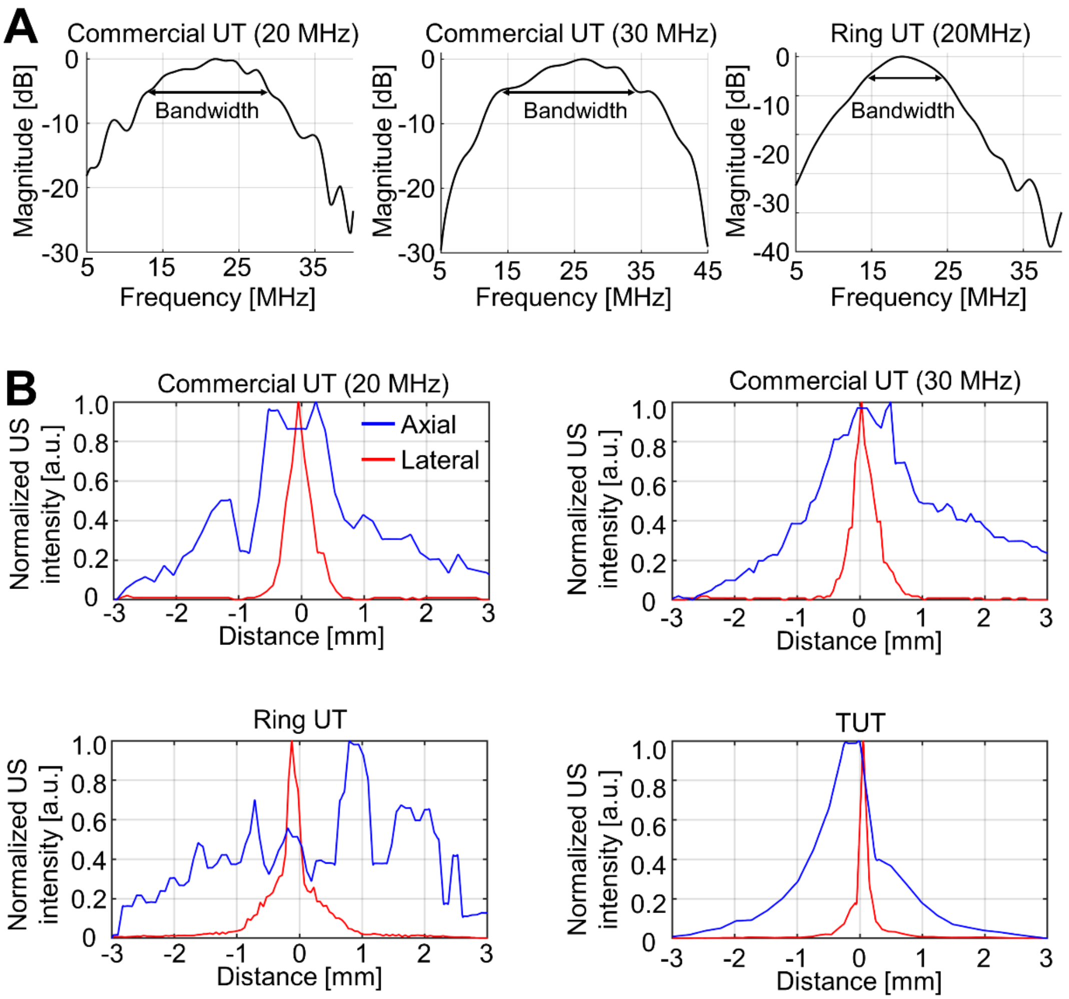

Fig. S1. (A) Frequency spectra of commercial UTs and ring UT. (B) Lateral and axial acoustic beam profiles of commercial UTs, ring UT, and TUT along the white dashed lines of the acoustic pressure fields in Fig. 2E. All UTs are spherically focused. UT, ultrasound transducer; US, ultrasound.

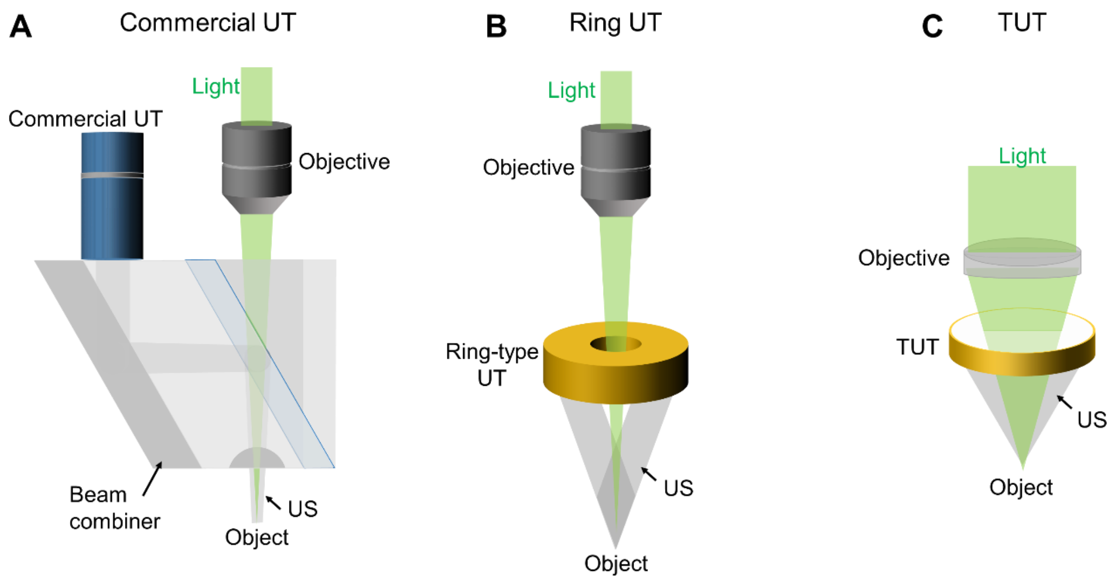

Fig. S2. Comparison of coaxial geometries that combine optical and ultrasound foci by using (A) an opto-ultrasound combiner with a commercial ultrasound transducer (UT), (B) a ring UT, and (C) a transparent ultrasound transducer (TUT). US, ultrasound.

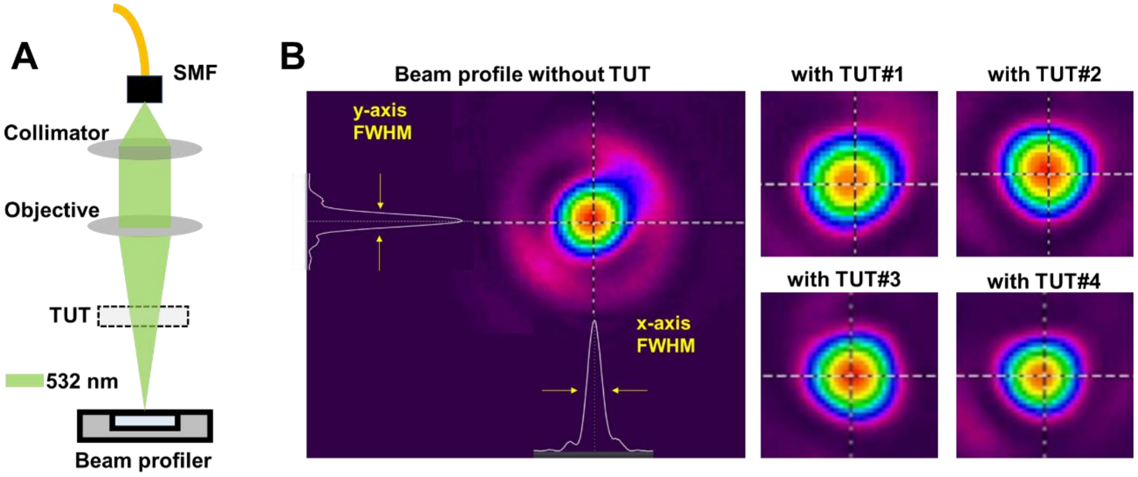

C

		W/O TUT	W/ TUT#1	W/ TUT#2	W/ TUT#3	W/ TUT#4
FWHM [μm]	X-axis	2.2	2.4	2.4	2.3	2.2
	Y-axis	2.3	2.5	2.5	2.4	2.5

Fig. S3. Analysis of measured beam profiles with the transparent ultrasound transducer (TUT). (A) Schematic of optical components and beam profiler. The 532 nm beam output from the single mode fiber (SMF) is collimated and then focused through the objective lens. (B) Beam profiles, measured before and after placing the TUT in the 532 nm beam path (N=4). (C) A table of full width at half maximum (FWHM) values of the beam profiles. The FWHMs of the beam profile without the TUT are 2.2 μm and 2.3 μm for the X and Y axes, respectively, but those with the TUT are 2.32 ± 0.01 μm and 2.47 ± 0.05 μm, respectively.

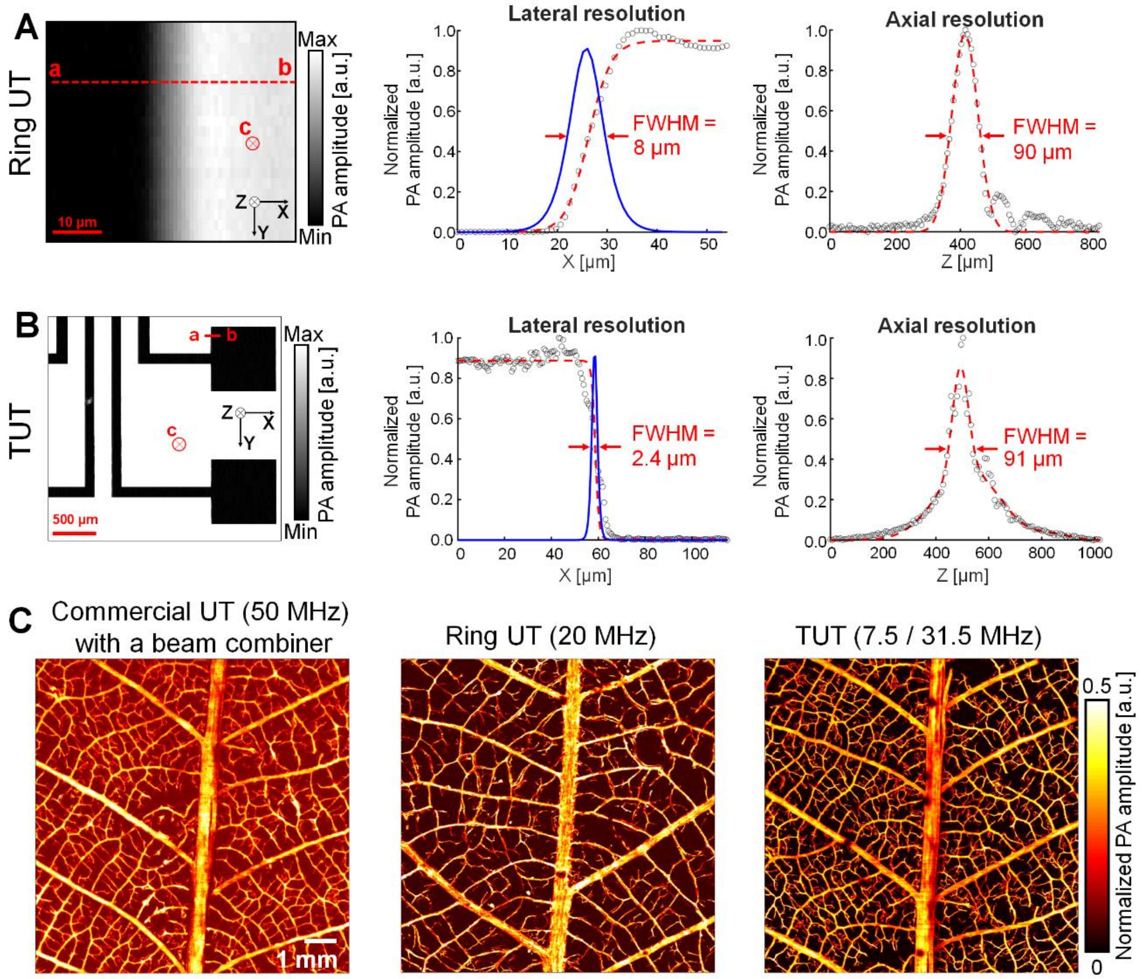

D

	Commercial UT (50 MHz)	Ring UT (20 MHz)	TUT (7.5 / 31.5 MHz)
Lateral resolution (μm)	3[6]	8	2.4
Axial resolution (μm)	33[7]	90	91
SNR (dB)	26	29	41

Fig. S4. Spatial resolutions of the PAI systems with a 20 MHz ring UT and a TUT. PA MAP images of (A) a chrome target and (B) a gold micro pattern target were acquired by the ring-UT-PAI system and the TUT-PAI system, respectively. For the lateral resolutions, the ESFs were fitted to the experimental data along the line a – b in both MAP images. Then, the fitted LSF were calculated by the first derivative of the ESFs. For the axial resolutions, the LSFs were fitted to the experimental data at the point c in both MAP images. (C) Leaf skeleton leaf PA MAP images captured using a 50 MHz commercial UT with an opto-ultrasound beam combiner, a 20 MHz customized ring UT, and the TUT, all at the same laser power of 50 nJ. The three PA images were normalized and displayed using the same dynamic range. (D) Summarized spatial resolutions and SNRs. TUT, transparent ultrasound transducer; UT, ultrasound transducer; MAP, maximum amplitude projection; LSF, line spread function; ESF, edge spread function; FWHM, full width at half maximum; SNR, signal-to-noise ratio; PA, photoacoustic; PAI, photoacoustic imaging.

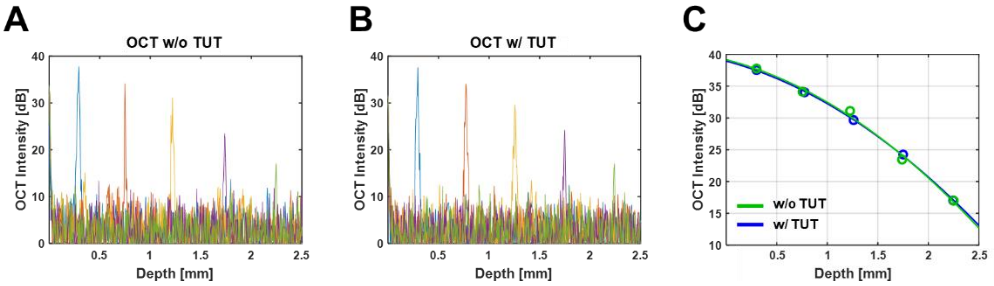

Fig. S5. Comparison of the roll-off measurements in an OCT system (A) without and (B) with a TUT. (C) Comparison of OCT peak intensities without and with the TUT. The illuminating light intensities on the sample, with and without the TUT, were made identical by compensating for the light attenuation by the TUT. OCT, optical coherence tomography; TUT, transparent ultrasound transducer**.**

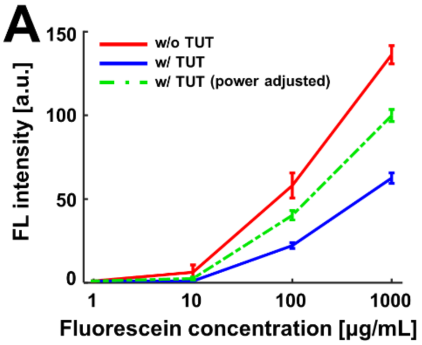

B

		Laser power (mW)	
w/o TUT		**0.50**	
		Laser power before passing the TUT	Laser power after passing the TUT
w/ TUT		0.50	**0.32**
		0.79	**0.50**

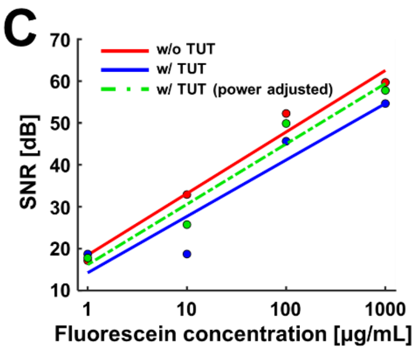

Fig. S6. Fluorescence (FL) sensitivity measurement. (A) FL sensitivity of fluorescein in a tube at 488 nm, without and with the transparent ultrasound transducer (TUT). The blue solid line (with the TUT) used the same laser output power as the red solid line (without the TUT). In the case of the green dotted line (with the TUT), the incident laser power on the sample was matched to that of the w/o TUT case by increasing the laser output power. (B) Comparison of laser power values measured before and after passing the TUT. The yellow highlighted values are the laser power irradiated on the sample for each case. (C) Comparison of FL SNRs with and without TUT.

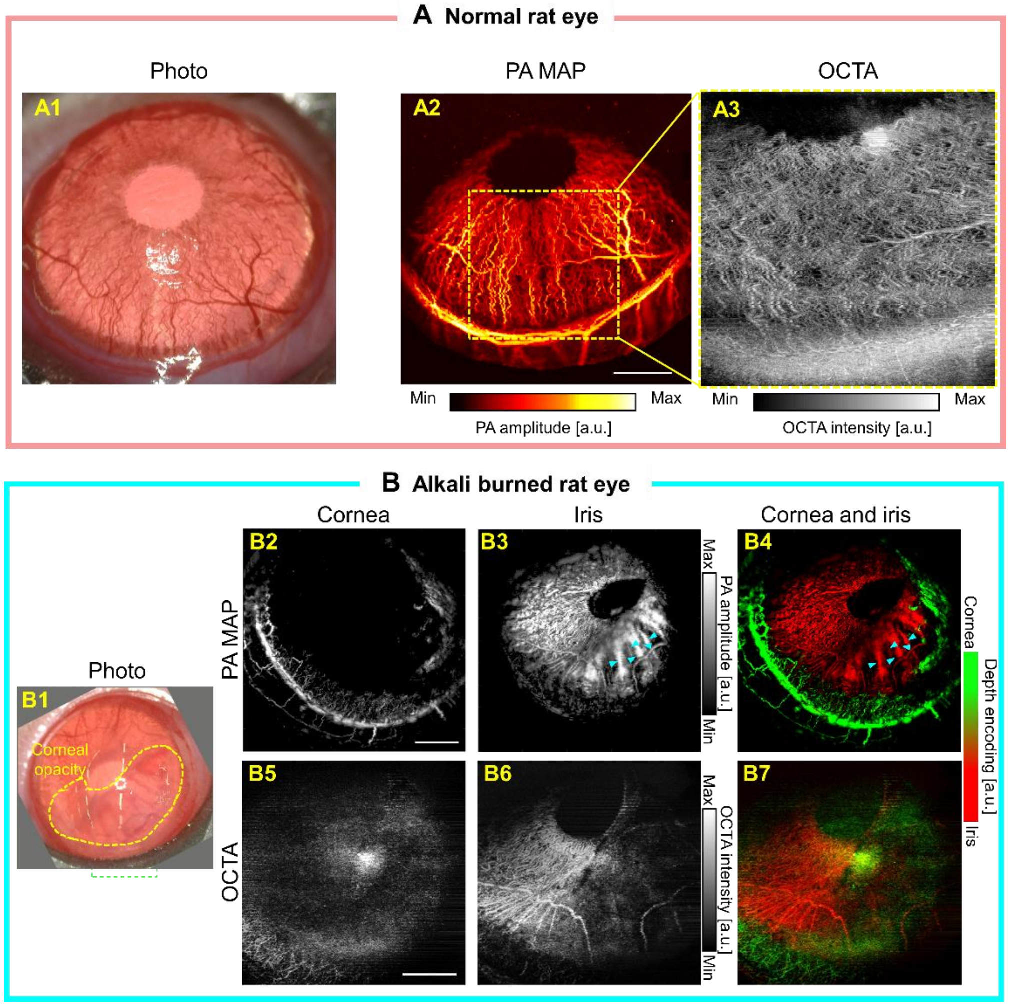

Fig. S7. *In vivo* PAI and OCTA of rats' eyes before and after alkali burns. (A) Photograph (A1), PA MAP (A2), and OCTA MIP (A3) images of normal rats' eyes. (B) After alkali burns, photograph (B1), PA MAP (B2–B4), and OCTA MIP (B5–B7) images. The cyan triangles point to iris blood vessels visible only in PAI (B3). Scale bar = 1 mm. PA, photoacoustic; MAP, maximum amplitude projection; OCTA, optical coherence tomography angiography, MIP, maximum intensity projection.

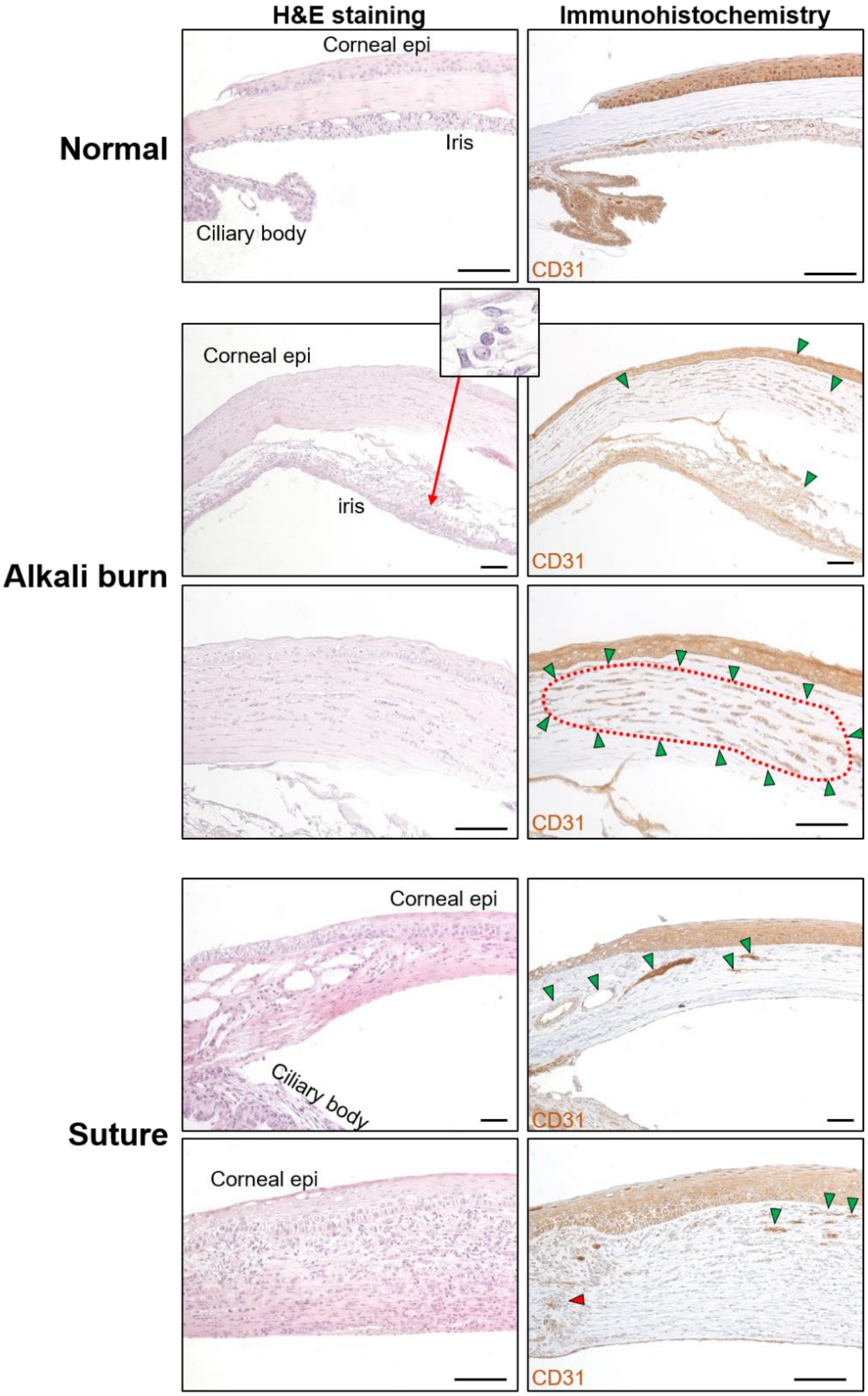

Fig. S8. Cross-sectional images of H&E staining and immunohistochemistry (CD31) in normal, alkali burn, and suture injured rat eyes. The CNVs after alkali burn and suture injury are identified by CD31 expression (green triangles). The red triangle indicates the position of suture needle. Scale bar = 100 μm. H&E, hematoxylin and eosin; CNV, corneal neovascularization; and Epi, epithelium.

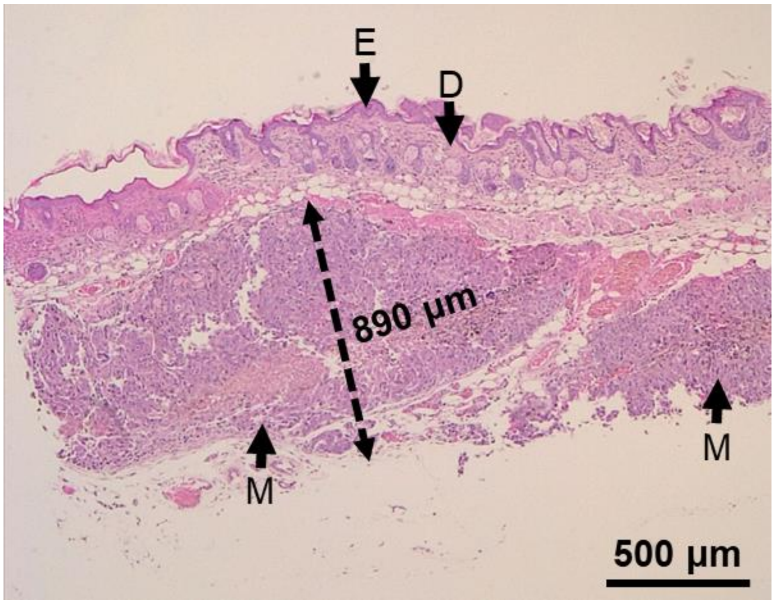

Fig. S9. A cross-sectional histopathology image of B16 melanoma. The measured thickness of the melanoma is 890 µm. E, epidermis; D, dermis; and M, melanoma.

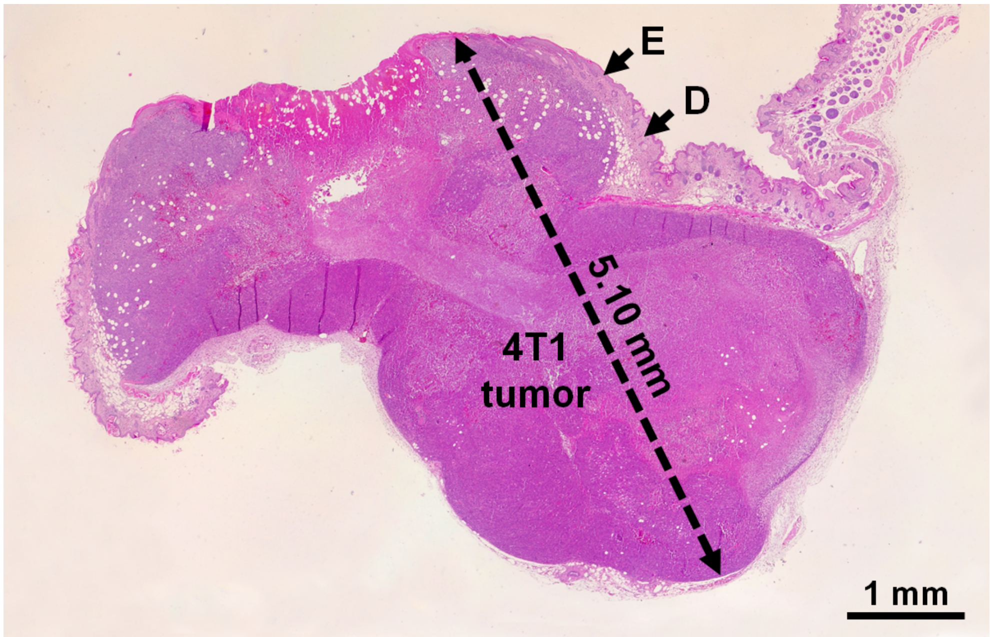

Fig. S10. A cross-sectional histopathology image of 4T1 breast carcinoma tumor. The measured thickness of the tumor is 5.10 mm. E, epidermis; and D, dermis.

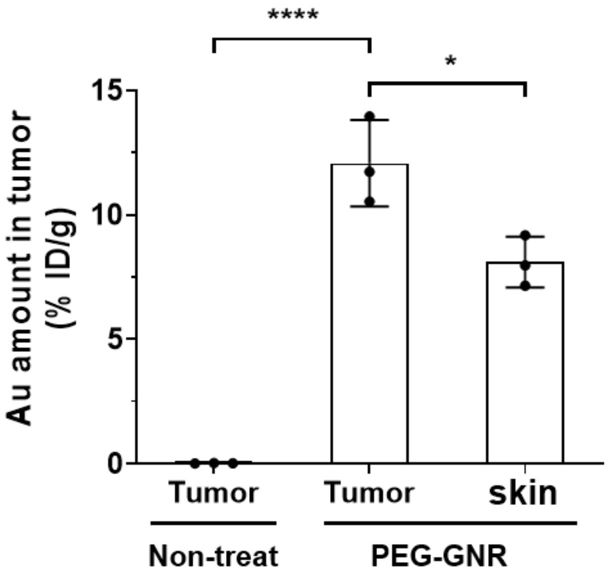

Fig. S11. ICP-MS results of Au in the 4T1 tumor and skin of mice at 8-hr after intravenous injection of the PEG-GNRs. Statistical differences were analyzed by performing one-way ANOVA. Data represent the mean ± SD (*P < 0.05, **P < 0.01, ***P < 0.001, ****P < 0.0001). ICP-MS, inductively coupled plasma mass spectrometry; PEG-GNR, PEGylated gold nanorod; hr, hours and SD, standard deviation.

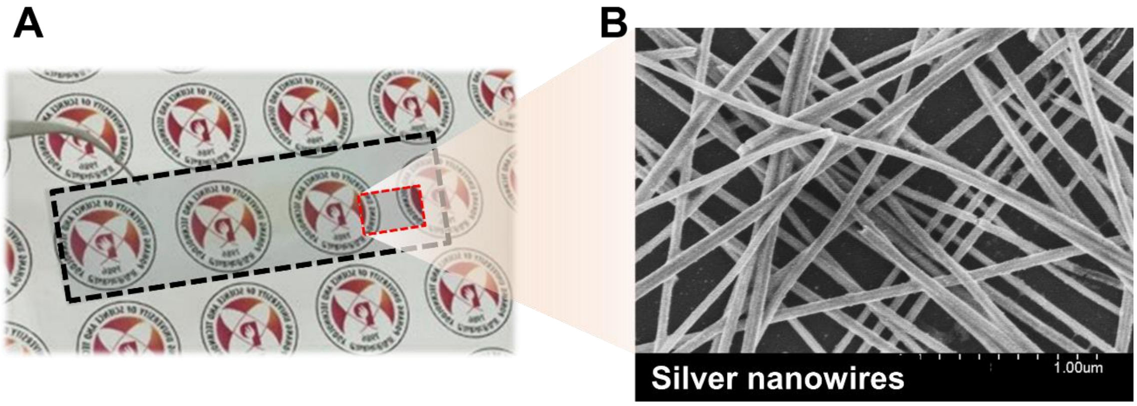

Fig. S12. Photographs of the AgNWs. (A) Transparent AgNWs-coated slide glass on the logo (indicated by the black dashed box). (B) Microphotograph of AgNWs within the the red dashed box in panel (A). AgNWs, silver nanowires.

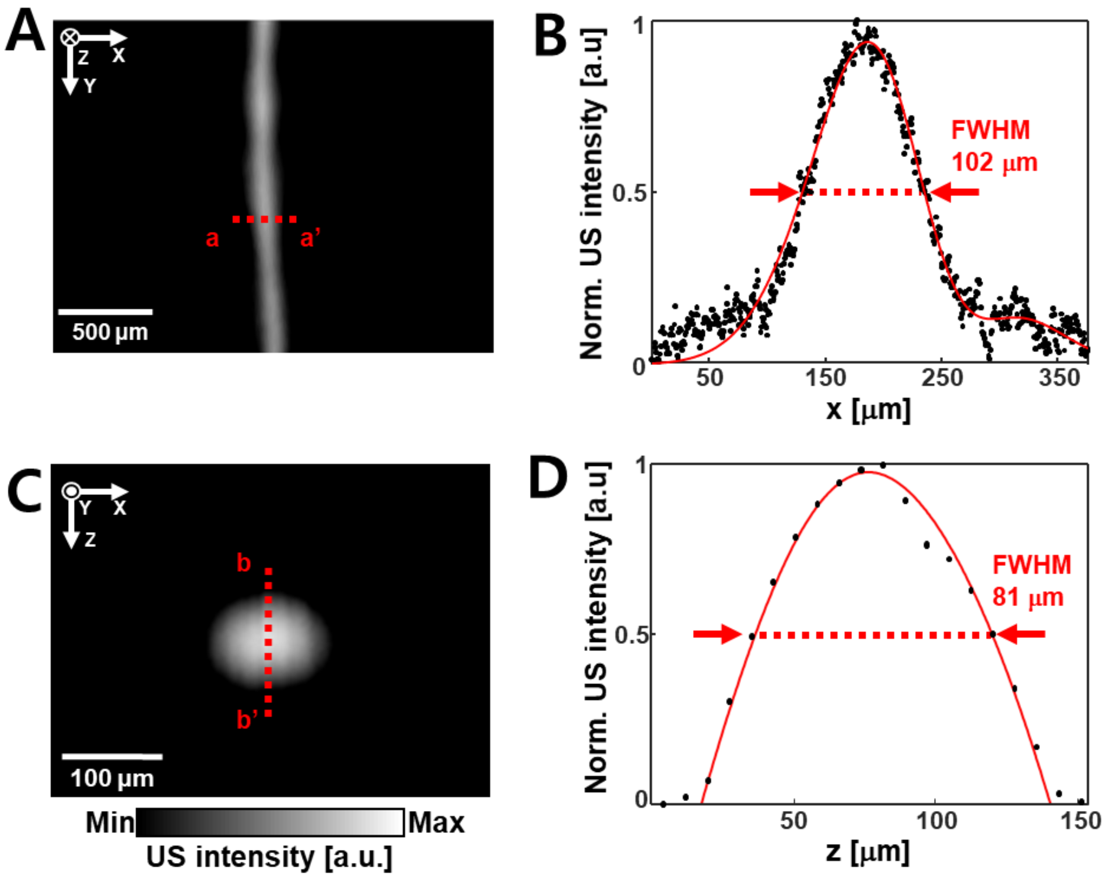

Fig. S13. Spatial resolutions of the USI system with a TUT. (A) US MIP image of a carbon fiber. (B) LSF fitting from the experimental data across the line a-a' in the panel a. The lateral resolution is 102 μm. (C) Cross-sectional US B-scan image along the line a-a' in the panel a. (D) LSF fitting from the experimental data across the line b-b' in the panel c. The axial resolution is 81 μm. USI, ultrasound imaging; TUT, transparent ultrasound transducer; FWHM, full width at half maximum; US, ultrasound; MIP, maximum intensity projection; and LSF, line spread function.

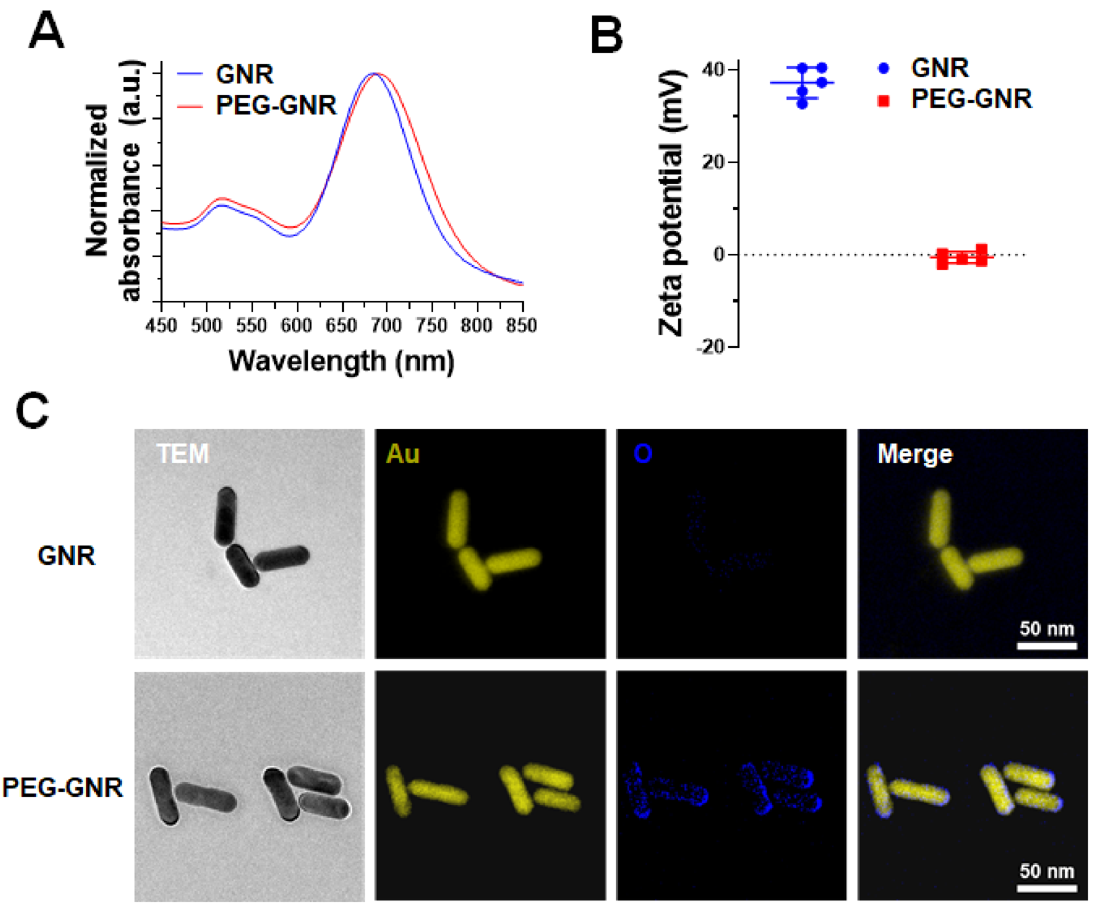

D

	λ_{max} (nm)	Length (nm)	Width (nm)	Aspect ratio	Zeta (mV)
**GNR**	686	46.1±3.0	16.7±0.6	2.75	37.3±3.3
**PEG-GNR**	691	46.2±4.2	16.5±1.4	2.80	- 0.57±1.3

Fig. S14. Characterization of gold nanorod (GNR) and PEGylated gold nanorod (PEG-GNR). (A) Absorbance spectra and (B) zeta potential of GNR and PEG-GNR. (C) High resolution TEM images of GNR and PEG-GNR. Oxygen is found only on the PEG-GNR surface, indicating a successful conjugation of the PEG to the GNR surface. (D) Summary of characteristics of the GNR and the PEG-GNR. TEM, transmission electron microscope; and O, oxygen.

Table S1. Imaging specifications for individual systems. USI, ultrasound imaging; PAI, photoacoustic imaging; OCT, optical coherence tomography; OCTA, optical coherence tomography angiography; and FLI, fluorescence imaging.

	USI	PAI	OCT	OCTA	FLI
Ophthalmology					
Field-of-view	5×5×3.8 mm^3	5×5×3.8 mm^3	6×6×3 mm^3	4.2×4.2×3 mm^3	6×6 mm^2
Axis	X-Y-Z	X-Y-Z	X-Y-Z	X-Y-Z	X-Y
Data acquisition time	9 min 17 sec	9 min 17 sec	40 sec	2 min 20 sec	Real-time
Oncology					
Field-of-view	15×10×7.7 mm^3	15×10×3.8 mm^3	4.8×4.8×3.4 mm^3	-	-
Axis	X-Y-Z	X-Y-Z	X-Y-Z	-	-
Data acquisition time	18 min 6 sec	18 min 6 sec	32 sec	-	-

min, minutes; and sec, seconds.

Movie S1 (separate file). *In vivo* 3D photoacoustic imaging of a rat's eye before and after alkali burn.

Movie S2 (separate file). *In vivo* 3D optical coherence tomography of a rat's eye before and after alkali burn.

Movie S3 (separate file). *In vivo* 3D ultrasound imaging of a rat's eye before and after alkali burn.

SI References

1. Park B*, et al.* (2020) 3D Wide-field Multispectral Photoacoustic Imaging of Human Melanomas In Vivo: A Pilot Study. *Journal of the European Academy of Dermatology and Venereology*.
2. Oraevsky A*, et al.* (2018) Clinical optoacoustic imaging combined with ultrasound for coregistered functional and anatomical mapping of breast tumors. *Photoacoustics* 12:30-45.
3. Jokerst JV, Cole AJ, Van de Sompel D, & Gambhir SS (2012) Gold nanorods for ovarian cancer detection with photoacoustic imaging and resection guidance via Raman imaging in living mice. *ACS nano* 6(11):10366-10377.
4. Lee J, Jeong C, & Kim WJ (2014) Facile fabrication and application of near-IR light-responsive drug release system based on gold nanorods and phase change material. *Journal of Materials Chemistry B* 2(47):8338-8345.
5. Orendorff CJ & Murphy CJ (2006) Quantitation of metal content in the silver-assisted growth of gold nanorods. *The Journal of Physical Chemistry B* 110(9):3990-3994.
6. Jeon S, *et al.* (2017) In vivo photoacoustic imaging of anterior ocular vasculature: a random sample consensus approach. *Scientific reports* 7(1):1-9.
7. Park, J, *et al.* (2016) Delay-multiply-and-sum-based synthetic aperture focusing in photoacoustic microscopy. *Journal of biomedical optics* 21.3: 036010.
